# An empirical study of the systemic and technical migration towards microservices

**DOI:** 10.1007/s10664-023-10308-9

**Published:** 2023-05-22

**Authors:** Hamdy Michael Ayas, Philipp Leitner, Regina Hebig

**Affiliations:** 1 Chalmers University of Technology and University of Gothenburg, Lindholmsplatsen 1, Kuggen-351, Gothenburg, 417 56 Sweden; 2 University of Rostock, Albert-Einstein-Straße 22, Rostock, Germany

**Keywords:** Microservices, Migrations, Grounded theory, Empirical research

## Abstract

**Context:**

As many organizations modernize their software architecture and transition to the cloud, migrations towards microservices become more popular. Even though such migrations help to achieve organizational agility and effectiveness in software development, they are also highly complex, long-running, and multi-faceted.

**Objective:**

In this study we aim to comprehensively map the journey towards microservices and describe in detail what such a migration entails. In particular, we aim to discuss not only the technical migration, but also the long-term journey of change, on a systemic level.

**Method:**

Our research method is an inductive, qualitative study on two data sources. Two main methodological steps take place – interviews and analysis of discussions from StackOverflow. The analysis of both, the 19 interviews and 215 StackOverflow discussions, is based on techniques found in grounded theory.

**Results:**

Our results depict the migration journey, as it materializes within the migrating organization, from structural changes to specific technical changes that take place in the work of engineers. We provide an overview of how microservices migrations take place as well as a deconstruction of high level modes of change to specific solution outcomes. Our theory contains 2 modes of change taking place in migration iterations, 14 activities and 53 solution outcomes of engineers. One of our findings is on the architectural change that is iterative and needs both a long and short term perspective, including both business and technical understanding. In addition, we found that a big proportion of the technical migration has to do with setting up supporting artifacts and changing the paradigm that software is developed.

## Introduction

Migrations towards Microservices Based Architectures (MSAs) are long and complex endeavours, that entail significant changes in the ways that systems of organizations are structured and operated (Newman , 2019). The complexity of migrations comes from the distributed nature of designing and developing MSAs (Zimmermann , 2017b; Newman , 2015), but also the multidimensional nature of migrations (Michael Ayas et al. , 2021a; Waseem et al. , 2021). Hence, empirically investigating migrations comprehensively from different points of view is important to understand their details (Di Francesco et al. , 2018). Guidelines, both originating from research and industrial best practice, can help organizations with microservices migrations (Newman , 2019; Balalaie et al. , 2016).

However, most practical and theoretical guidance focuses on isolated aspects of the migration (e.g., how to technically split off services). Hence, it is not always clear how these disparate aspects of migrations connect to each other. On the one hand, microservices migrations are characterized by overarching patterns such as decomposing the old system or introducing new technologies (Balalaie et al. , 2018). On the other hand, these overarching patterns are operationalized by practical activities that are more specific and narrow in scope (Fritzsch et al. , 2019; Knoche and Hasselbring , 2018). What we are lacking is a clear overarching view on how precedent MSAs migrations are conducted, and which activities on organizational and technical levels are included (Di Francesco et al. , 2019). For example, migrations entail technical activities that actually take place in the code (e.g. actually splitting the code-base into small services) (Gysel et al. , 2016), but also activities that are structural and relevant to how organizations operate (Hassan et al. , 2020).

In this study, we address this gap through empirical, qualitative research in which we focus out from initiatives that take place in isolation. Instead, we present how MSAs migration initiatives connect in an overarching journey. The objective is to present what the overall journey towards MSAs entails, who is responsible for the different initiatives and when different parts of these initiatives take place. Hence, we separate the activities that migrating organizations carry out into a set of guidelines for migrations to microservices.

We do so by conducting an interview study with 19 engineers from 16 case companies of different size, industries, and geographical regions. All interviewed developers have been part of their companies’ migration journeys towards microservices. Following that, we evaluate and extend the activities of the aggregated migration journey with more empirical data using questions and answers from StackOverflow. We conduct manual, qualitative analysis of 215 relevant posts, also using techniques from grounded theory. As a result, we provide an in-depth characterization of MSA migration activities including concrete solutions. The research questions that guide this study are as follows:

***RQ1*** *: How are microservice migrations structured?*

Our results showcase that microservices migrations are an iterative process, and take place in two modes of change, *1) the systemic and 2) the technical* migration. Change on the systemic migration is on a long-term and strategic scope, whereas change on the technical migration is on a short-term scope and is repeated in frequent migration sprints.

***RQ2*** *: What activities do the systemic and technical modes of change entail?*

We identify in total 14 activities that happen in the 2 modes of change. The systemic migration has 6 activities that drive the overall direction of the migration and are taken by management, software architects and senior engineers. Activities in the systemic migration result in big, structural changes on the overall system. The technical migration has 8 activities with a smaller scope, on the technological changes. Activities in the technical migration result in a different implementation of a given technical artifact.

***RQ3*** *: What common solutions are available to realize these activities?*

This study organizes and presents 53 different solution outcomes that are used by engineers to realize the migration. The systemic migration has 20 solution outcomes that are the concrete results of the work of management, architects and senior engineers when migrating. The technical migration has 33 resulting solution outcomes that engineers devised in their migrations.

The results to the aforementioned research questions result to a set of guidelines for migrations to microservices. Therefore, in this paper we argue that:

- Our empirical analysis can give practitioners the expected migration journey, and can serve as a road-map to what activities such a journey entails.
- We provide a high-level model of the migration journey that can help plan and structure migrations as well as share a common picture of the state of a migration.
- The resulting journey entails a set of specific guidelines in the form of activities and solution outcomes for each part of the system that can help organizations develop a framework on which they can base their migration.
- Our results also provide a detailed theoretical basis to researchers for investigating further the different aspects of microservices migrations.

An earlier version of this research has appeared in a previous conference paper (Michael Ayas et al. , 2021b). The current study builds on the modes of change and activities already identified in the previous paper, but is extended by a second study step (analysis of StackOverflow data), leading to deeper insights into concrete solution outcomes within all activities of a microservice migration.

## Related Work

Our main area of study are migrations towards microservices. In the following we briefly summarize the current state of research, and discuss what gaps we address in our work.

### Benefits of Microservices

Microservices are a way of structuring systems into loosely coupled pieces that are developed and operated independently, each with its own individual resources (Thönes , 2015). These individual pieces communicate with each other to compose a complete system (Zimmermann , 2017b). There are many fundamental differences between a software application based on a monolithic architecture and a system based on microservices (Newman , 2019). However, we still need to understand more in detail these differences and how the transition between the two architectures can possibly take place.

Migrating to a microservice-based architecture can be very rewarding for organizations because microservices promise to enable many benefits (Newman , 2019; Singleton , 2016; Soldani et al. , 2018). The migration journey is often very helpful to improve the developed system (Balalaie et al. , 2016) and decrease technical debt on the long term (Lenarduzzi et al. , 2020). Hence, organizations are very often extremely motivated to migrate towards microservice-based architectures and there are many potential ways to do so (Newman , 2019; Mazlami et al. , 2017). Microservices promise improvements in many aspects, such as scalability, maintainability and continuous development (Di Francesco et al. , 2018). Specifically, resource-demanding parts of a software application can be scaled independently and unburden the rest of the system. Also, the modular organization of the system with minimal dependencies allows improved maintainability (Dragoni et al. , 2017). In addition, the flexibility in service design that can be achieved enables a lot of potential in continuous delivery of new business value (Dragoni et al. , 2018). All these benefits are promised for the completion of the migration. However, it is not clear how they can be achieved in intermediary stages and have tangible benefits during the migration.

### Migrating towards microservices

Microservices are increasingly getting widely adopted by different organizations. Usually there is a large leap between a monolith and a microservice architecture (Newman , 2015). A migration / transition is the crucial project that takes the software development organization through the leap (Hassan et al. , 2020; Taibi et al. , 2017). Research on microservice migration projects gains popularity (Hassan et al. , 2020). Previous research investigated the area surrounding the architectural characteristics of microservices migrations (Balalaie et al. , 2018; Fritzsch et al. , 2019), as well as how it relates to the overall development process (Taibi et al. , 2017). In addition, existing research provides several solutions on how to technically enact and facilitate microservices migrations (Waseem et al. , 2020). These solutions include splitting a system, transforming the code of an application, identifying services in a monolith (Fritzsch et al. , 2018) or decentralizing their data management and governance (Loukiala et al. , 2021). Also, such solutions often provide tools on how to identify and decompose services, assuming a technical and deterministic viewpoint on the migration (Gysel et al. , 2016). This is not always ideal, as migrations are more often than not complex endeavours with many things to consider (Newman , 2019; Waseem et al. , 2021) – so many that it seems taunting to perform a migration in a one-off project (Taibi and Lenarduzzi , 2018).

In practice, researchers have observed that most microservice migrations entail weaknesses or limitations that stem from insufficient migration planning, leading to Microservices Bad Smells (Taibi and Lenarduzzi , 2018) or Anti-Patterns (Taibi et al. , 2020). To prevent such bad smells, Balalaie et al. (2018) provide a valuable set of patterns that can guide a microservices migration initiative (Balalaie et al. , 2018). In addition, Auer et al. (2021) present a metrics-based assessment framework for transitioning systems to microservices (Auer et al. , 2021). Current research also provides sufficient arguments and decision models on how to tackle problems that arise and how to technically perform a migration (Waseem et al. , 2021). However, all those lead to work for engineers that needs to be done and more often than not, the required solutions to implement them are unknown, in order to be planned accordingly. Also, even though existing work organizes rigorously and systematically aspects that describe architecting systems with microservices (Di Francesco et al. , 2019), there is a gap in studying with similar rigour migrations specifically.

### Architectural Migration

Our work also builds on a body of work on architectural migrations of legacy systems (often to service- or cloud-based architectures). Early work during the advent of service-oriented architecture often focused on identifying (or “incubating”) services in a legacy system Zhang and Yang (2004). This problem also re-appears when migrating to microservices, particularly when defining criteria for decomposition (Section 4.2.2).

There already exists significant research on how to conduct architectural migrations to cloud systems Zimmermann (2017a); Ahmad and Babar (2014). Many of the solutions identified in these works also appear during microservice migrations, and are often applied by practitioners in this context. Particularly, Jamshidi et al. have previously identified cloud migration patterns, which, to a large extent, are also applicable to the migration to microservices Jamshidi et al. (2014). In addition, there is extensive literature describing and analyzing different strategies for migrating legacy systems to the cloud in general Zhao and Zhou (2014). Existing research presents the general challenges of the general migration process towards the cloud Gholami et al. (2017) and there are approaches that can support refactoring of systems (towards Platform-as-a-Service Borges et al. (2018) or the modernization process and roadmap towards the cloud Jain and Chana (2015).

### Tools and Technology

The technologies and tools that implement microservices have grown over the years and get applied extensively (Hassan et al. , 2020). However, techniques to evaluate decomposition approaches are often not evaluated on applications from industry, making problems appear in later stages of the development lifecycle, i.e., in production, when applied (Di Francesco et al. , 2019). This improves with recent research on empirically analysing real-world microservices systems (Camilli and Russo , 2022) and their traces (Zhou et al. , 2021). However, the focus is mainly on what stakeholders and developers could do differently and not what stakeholders and developers face during a migration. For example, migrating towards microservices entails challenges and activities that are not always in line with current best practices and these can be identified and investigated (Di Francesco et al. , 2018; Taibi and Lenarduzzi , 2018). Hence, learning from organizations that migrated to microservices, can also help us understand the elements of a migration journey and raise awareness for them over to future migrations.

## Methodology

**Fig. 1:**
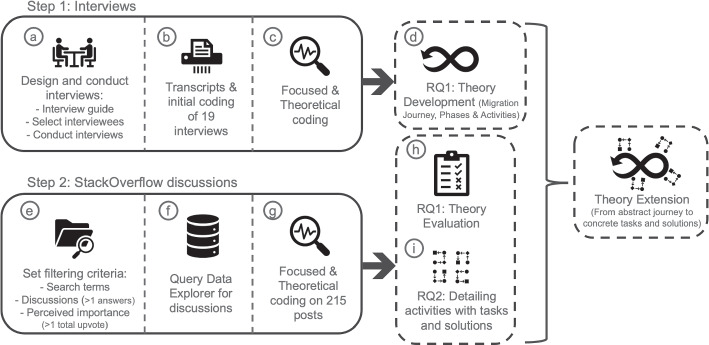
An overview of the followed research methodology

Our research method is a mixed methods study, consisting of two main methodological steps as shown in Fig.  1.

First, we conducted interviews with practitioners who have recently participated in microservices migration projects. An analysis of the interviews revealed different modes and activities of the migration journey.

In a second step, we analyzed 215 posts from the Q &A website StackOverflow. The objective of analyzing the posts is to evaluate the results from the interviews and further deepen our knowledge on the identified activities, by identifying involved solution outcomes.

Combining both, interviews and posts from StackOverflow, allows us to get a more holistic picture of what developers go through during a migration journey. On the one hand, the interviews served well to assess how practitioners perceive and experience their journey. On the other hand, the discussions from StackOverflow allowed us to identify: *i) the critical points in the journey that prompt practitioners to reach out to others to deliberate* and *ii) the detailed technical solutions that practitioners used throughout the journey*. Furthermore, the posts that we analyze helped to empirically identify common solutions that can be associated with the activities. Thus, the combination of both data sources, interviews and StackOverflow posts, allowed us to gain a more complete picture, by combining insights from practitioners’ perceptions when reflecting on the journey with traces of the discussions they created during their respective journeys.

The analysis of both, interviews and posts, relies on techniques found in Grounded Theory (GT) (Charmaz , 2014), namely coding, memoing, sorting and constant comparison. Based on guidelines for GT in software engineering research, we cannot claim to use the classic GT method. Instead, we used an adaptation of constructivist GT as we had significant previous exposure to literature prior to the study (Stol et al. , 2016), such that some of our themes align with both, previous research (Balalaie et al. , 2018; Hassan et al. , 2020) as well as commonly identified processes (Newman , 2019).

### Interviews

We conducted semi-structured interviews (Fig. 1 methodological Step 1). The interview guide, was constructed based on our first research question. However, we gave participants significant freedom to describe their own migration journeys in their own words. In accordance to constructivist GT, we started with an initial research question that evolved throughout the study (Stol et al. , 2016). The initial research question was inspired both from practical experience and literature on the subject. At start, we targeted to address more generally the underlying elements of a microservices migration, but early on we needed to narrow the scope and focus specifically on the migration process.

It is worth noting that the interviews are collected in another study with focus on decision-making during microservices migrations (Michael Ayas et al. , 2021a). Even though both studies rely on the same interviews, we conducted a new analysis for this work, based on a different portion of the data. The main methodological difference in this study is that saturation is observed retrospectively with the available data, rather than driving the termination of inviting more participants.

The interview guide can be found in our replication package (Michael Ayas et al. , 2022). We omit interview transcripts from the replication package to preserve interviewee privacy and protect potential commercial interests of our interviewee’s employers.

#### Participants

We relied on purposive sampling (Baltes and Ralph , 2020) and our personal network (e.g., through current and previous projects, colleagues, or students) to design and conduct the interviews (Fig. 1, sub-step (a)). The recruited interview participants have a rich repertoire of experiences with microservice migrations. Furthermore, we used a snowballing approach, where we asked each interviewee to refer us further to other potential participants from their networks. This way we tackled the well-known challenge of recruiting a sufficient number of engineers for interview studies. The selection criteria for participants during the study were adjusted in order to include more experienced engineers along the way. We used an adapted saturation approach (Charmaz , 2014) in which we judged during data analysis that no new insights were appearing and therefore, there was no need to continue inviting more participants. For the selection of interviewees and case organizations, we used a set of acceptance criteria. Specifically, our interviewees are *(a)* software engineering professionals (not students) who *(b)* have participated (or were close observers) in a microservice migration project within their professional work. An overview of the participants is found in Table 1.

We have interviewed 19 professionals from 6 different countries (Cyprus, UAE, Germany, Romania, Sweden, The Netherlands), of which 18 were male and one female. Interviewers had on average 7.5 years of experience (ranging from 2 to 21) and they have worked at medium to large companies in twelve business domains. Note that some of the interviewees have 2 or less years of experience with MSAs. We consider that to be representative of companies that are still in process of migrating towards microservices architectures. In addition, the migration cases are about systems delivered to external customers (e.g. Enterprise SaaS), in-house enterprise solutions for internal users and also Software Applications sold as a service (e.g. mobile app). Each interviewee worked in (at least) one case of microservices migration and we consider migrations from 16 different companies, as shown in Table 1.

**Table 1:** Interview participants and case organizations. Organizations size is reported in approximate numbers of full time employees

*Organization*	*Org. Size*	*Industry*	*Interview*	*Role*	*Experience (MSA)*
Org1	50	Enterprise SaaS	I1	Full stack developer	2 (1)
Org2	4,000	Gaming	I2	Software Engineer	2 (2)
Org1	50	Enterprise Software	I3	Senior Team Leader	12 (2)
Org3	36,000	Banking Systems	I4	Software Engineer	2 (1)
Org4	9,000	Banking Software	I5	Software Engineer	19 (2)
Org1	50	Enterprise Software	I6	Software Engineer	2 (1)
Org5	3,000	Aviation Software	I7	Software Engineer	7 (2)
Org6	30,000	Telecommunications	I8	Software Developer	3 (3)
Org7	27,000	Enterprise Software	I9	Computer Scientist	5 (5)
Org8	200,000	Cloud Computing	I10	Principal Software Engineer	7 (4)
Org9	33,000	Marketing Analytics	I11	Software Engineer	6 (3)
Org10	150	Healthcare Software	I12	Data Engineer	6 (2)
Org11	83,000	Cloud Computing	I13	Senior Cloud Architect	10 (5)
Org12	50	Energy Software	I14	Software Engineer	4 (1.5)
Org12	50	Energy Software	I15	Software Architect	4 (4)
Org13	30	Logistics / Planning	I17	Co-founder	8 (5)
Org14	62,000	Logistics / Planning	I16	Software Architecture Consultant	13 (4)
Org15	25	Manufacturing	I18	CTO	10 (6)
Org16	1m	Manufacturing	I19	Enterprise Architect	21 (5)

Experience is in years and values in brackets are on experience with microservices

#### Protocol

We conducted our interviews over a period of six months. Each interview took between 30 and 60 minutes (Fig. 1, sub-step (a)). Due to the COVID-19 pandemic that was ongoing during our data collection, as well as geographical distance, interviews had to be carried out through video conferencing. Prior to each interview, participants were asked to sign a consent form, and consent to recording the interview. Further, participants were made aware that they can drop out of the study at any point, which no interviewee made use of. We did not offer financial rewards to study participants.

In order to learn about the migration journeys of the interviewees, we opted for a semi-structured interviewing process. The semi-structured format gave the study participants the freedom to describe their journey, as they experienced it. The prepared questions (can be seen in the replication package of this study Michael Ayas et al. (2022)) were used to maintain the scope of the discussion. The interviews were initiated using two to five introductory questions, depending on how elaborate the participant’s answers were. The introductory questions were used to obtain descriptions of the participant’s background, experience, role, industry, and type of software they migrated to microservices. Then, seven questions were prepared to guide participants in reporting about the migration journey they experienced (listed in Table 2). In addition, some clarification questions were also asked, to stretch on certain topics that the interviewees touched upon but did not go into depth.

**Table 2:** The interview guide used in the semi-structured interviews

***#***	***Question***
*Q0*	Background information (education, training, working experience, experience with microservices, current role, type of software)
*Q1*	What do you consider as microservice architecture? What defines it and what are you trying to get out of it?
*Q2*	Can you briefly describe your journey of architecting, designing, and implementing a microservices-based architecture while migrating from a monolith (migration process, transformation etc.)?
*Q3*	Can you tell me about different things/properties/aspects that you have considered before starting the design of the microservices architecture that enhanced your work (technical or organizational, internal or external)?
*Q4*	What did you do different than existing guidelines and why? Did you follow the guidelines completely, one-to-one?
*Q5*	Were there any problems/issues or surprises that came along? (regarding the way about things that you did not consider and afterwards you felt that you should have considered? What, why, how?)
*Q6*	What were the costs of facing the different problems and the aspects not considered? (use example from interview)
*Q7*	Can you describe a wish list of 3 things you would like to have in retrospective?

#### Analysis

We applied initial, focused and theoretical coding on the transcribed interviews (Charmaz , 2014; Stol et al. , 2016). After conducting every interview, it was transcribed and analyzed with initial coding (Fig. 1, sub-step (b)). During the initial coding we analyzed the data horizontally by fracturing them to find relevant statements. During the focused coding (Fig. 1, sub-step (c)), we aggregated and connected those excerpts into categories and themes, analyzing them vertically until achieving saturation. During the theoretical coding we specified the relationships of the connected categories and integrated them into a cohesive theory, by conducting both horizontal and vertical analysis. The initial coding was conducted by the first author. All three authors collaborated in focused coding in three card sorting and memoing sessions lasting three to four hours each.

In initial coding, the first author coded those statements from the interviews, where practitioners described their actions during the migration journey. It was observed in the first focused coding session that most of these statements were about actions that practitioners performed during a migration. These actions were responses either to issues that practitioners faced or to (non-)functional requirements that needed to be fulfilled for the migration. Consequently, in the focused coding sessions, the actions of practitioners were recorded and organized into activities and phases. The separation of different activities is based on what interviewees described as separate tasks or as tasks with a different nature of executing. In the following iterations of coding, descriptions of the activities were gathered from interviews and enriched with more information, also concerning the phases. In addition, the distinction of longer and shorter-term activities started becoming apparent and therefore, we sorted the activities and phases in two iterative modes of change. Finally, in the theoretical coding, the three authors reflected more in-depth to how the activities link together and the iterative perspective of the migration started developing.

All resulting findings are supported by statements from multiple participants. Finally, it is worth mentioning that literature played a supporting role to our analysis in order to enhance the validity of our findings. Existing literature helped us to understand more comprehensively the statements of software developers during the interviews in combination with the authors experiences and previous exposure to the topic. Also, in this analysis we took into account existing research guidelines on creating processes and taxonomies in Software Engineering (Ralph , 2019).

### Posts from StackOverflow

We analyse posts from StackOverflow (Fig. 1, methodological step 2) through the lens of extracting the solution outcomes that engineers make when migrating. StackExchange is a data source for mining data from Q &A websites and it is widely used in Software Engineering research (Tahir et al. , 2020; Papoutsoglou et al. , 2022; Wen et al. , 2021; Chen et al. , 2020). Such websites and forums that facilitate Q &A across software development communities are hosting a lot of information of software engineers’ rationale and day-to-day work (Baltes and Diehl , 2019). Examples of different Q &A websites are StackOverflow, Software Engineer, DB professionals (and many more). We conduct a purely manual analysis on the mined posts, with techniques from grounded theory. This allows us to capture contextual information and describe in detail activities and solutions that engineers make when migrating towards microservices. Also, we identify the potential courses of action in migrating towards microservices and enrich the already identified migration journey from the interviews.

This inductive approach in the analysis allows us to make observations from an additional source that contains more concrete details on what engineers do than the interviews (which discuss on a more abstract level the general approach).

#### Data Gathering

The first step, was to identify which sites and forums are in the scope of this study. Specifically, Stack Exchange at the time of writing maintains data from Q &A websites of 175 communities.^1^ Due to the authors knowledge on the topic from interviews, previous experiences and exposure to the relevant literature, we were able to identify that relevant topics such as *microservices, migrations, DevOps, architectural transitions and data decentralization* are most commonly discussed in the StackOverflow community. Hence, we limit our data collection to this community.

In the second step, we gathered data from Stack Exchange and created a data-set for analysis using SQL queries in the platform’s Data Explorer. The queries were designed to gathering questions that initiate discussions on the topic of microservices migrations (Fig. 1, sub-step (e)). Therefore, the keyword combinations *“microservice”* AND *“monolith, migration and transition”* were searched in the title or body of posts. The initial search resulted to 265 question posts. As acceptance criterion, we used only posts with a positive total voting score and which have received at least one answer, reducing the number of question posts to 76. Hence, we only include posts were the content is validated and discussed by at least one peer engineer, having potential to contain useful information. Also, the presence of answers allows us to get more information about concrete solutions and when we gathered the answers of the 76 questions our dataset formed into 215 posts in total.

**Table 3:** Descriptive statistics of the 76 questions and discussions under questions gathered for analysis

	*Average answers*	*Average words*	*Standard deviation*	*Users*
**Questions**	1.83	224	+/- 135 words	74
**Discussions**	-	354	+/- 282 words	141

The gathered data is of textual format and varies in length from post to post. Typically, questions entail descriptions that are 1-3 paragraphs long and the answers entail one to five posts, without a clear pattern in their size, as shown on Table 3. The resulting format of the posts from querying the Data Explorer was suitable for manual and in-depth analysis of the contents from each post.

As the third step, we gathered the answers of each question post. Therefore, we run a second SQL query (per question) in the Data Explorer to download the entire discussion (Figure 1, sub-step (f)). Each discussion includes all the answers that were linked to the respective question. All the resulting queries can be seen in our replication package (Michael Ayas et al. , 2022).

#### Data Pre-processing and Analysis

We imported the gathered posts into the tool Nvivo^2^, which is suitable for qualitative analysis of unstructured data. Doing so, we transform the query results from the CSV format to text files, creating one file per question. The answers for each question were added in a separate file. Answers to the same question were analysed together (also with the question at hand), in order to keep track of the entire discussion’s contextual information and topic.

For the analysis of the data we used the techniques of coding, sorting of codes and identifying patterns (Fig. 1, sub-step (g)). The starting point of this analysis was the migration journey derived from the interviews. Specifically, the first author read each question and created topical codes. Then, each code was assigned to one activity from the migration journey developed from the interviews. In this way, the theory about the migration journey developed from the interviews was evaluated with further empirical data (Fig. 1, sub-step (h)). Specifically, the posts confirmed the relevance of the previously identified activities.

Next, the first author read each answer of the question at hand and coded the potential solution outcomes, to enrich our knowledge about the activities of the migration journey (Fig. 1, sub-step (i)). The whole team held weekly meetings to discuss the allocation of codes to activities and deliberate on the content of the derived migration journey. In these weekly sessions, axial coding was performed in order to derive what activities practitioners discussed, based on the support that practitioners’ requested from the StackOverflow community. In addition, the contextual information of these activities, based on the details given in their posts, and the different solutions that were discussed in the posts were extracted.

This also led to modifications on the initially recorded migration journey. The modifications were along the lines of:

1. Clarifying the scope of each mode of change. For example, we specified that the systemic mode of change concerns mainly architects, management and senior engineers.
2. Clarifying the scope of the identified phases of the migration journey and merging them into three distinct phases. For example, we merged into one phase the initial phases of “Setting up supporting artifacts” and “Implementing technical overhead”.
3. Merging activities that included solutions with similar nature or with similar scope. For example, in the last phase of the systemic migration we merged the activities into one that contains multiple solution outcomes.

The boundaries of activities are defined with the following rules:

1. Grouping solution outcomes that have a similar contextual scope. Specifically, focusing on a specific part of the system (i.e. backend, frontend etc.) or focusing on the business aspect of changing to microservices (e.g. Clarifying migration drivers).
2. Grouping solution outcomes that have a similar working task scope. For example, setting up communication between services or addressing cross-cutting concerns throughout the system altering the software systematically in a similar way across services.

In our analysis, we first identified solution outcomes of each activity and then we inferred from the data whether the solution outcome is an alternative outcome (mutually exclusive) or not.

#### Resulting Changes to Initial Theory

Posts from StackOverflow contributed in identifying new information on detailing descriptions of activities and solutions. In addition, this step enriches the identified migration journey by altering some of the interview results. Specifically, the analysis of StackOverflow posts lead to merging the initially four derived phases of a migration iteration into three phases (described in the results section). In addition, some activities identified in the interviews were merged, resulting to the description of 14 activities (from initially 18). However, the most significant impact that StackOverflow posts have in the results is the identification and addition of 53 solution outcomes, describing the detailed internal elements of each activity. Consequently, the differences on this step are reconciled by extending in detail the descriptions of activities on the one hand, and scoping the focus of activities to specific solutions discussed in StackOverflow.

## Results

Our analysis results in a migration journey that materializes in different modes of change within the migrating organization. In this study, we describe the process that comprehensively covers the migration from structural changes to specific changes that take place in the work of engineers. Specifically, we first describe the overall organizational migration journey that takes place in two modes of change. We refer to modes of change to indicate that MSAs migrations take place at different levels of an organization. Modes of change can be structured in phases. In these phases, we specify the journey with migration activities, that a migrating organization performs. Migration activities are high-level actions that realize the migration. In addition, we further derive and aggregate solution outcomes. They concretely describe what is performed during a migration activity. We differentiate *1) potential solution outcomes* (nice-to-have or required designs) and *3) alternative solution outcomes* that are mutually exclusive. Note that of course not all identified activities will be executed during every migration project. Whether an activity needs to be executed depends on the infrastructure, requirements, and artifacts that are already in place.

### Overview of Migration Journey

**Fig. 2:**
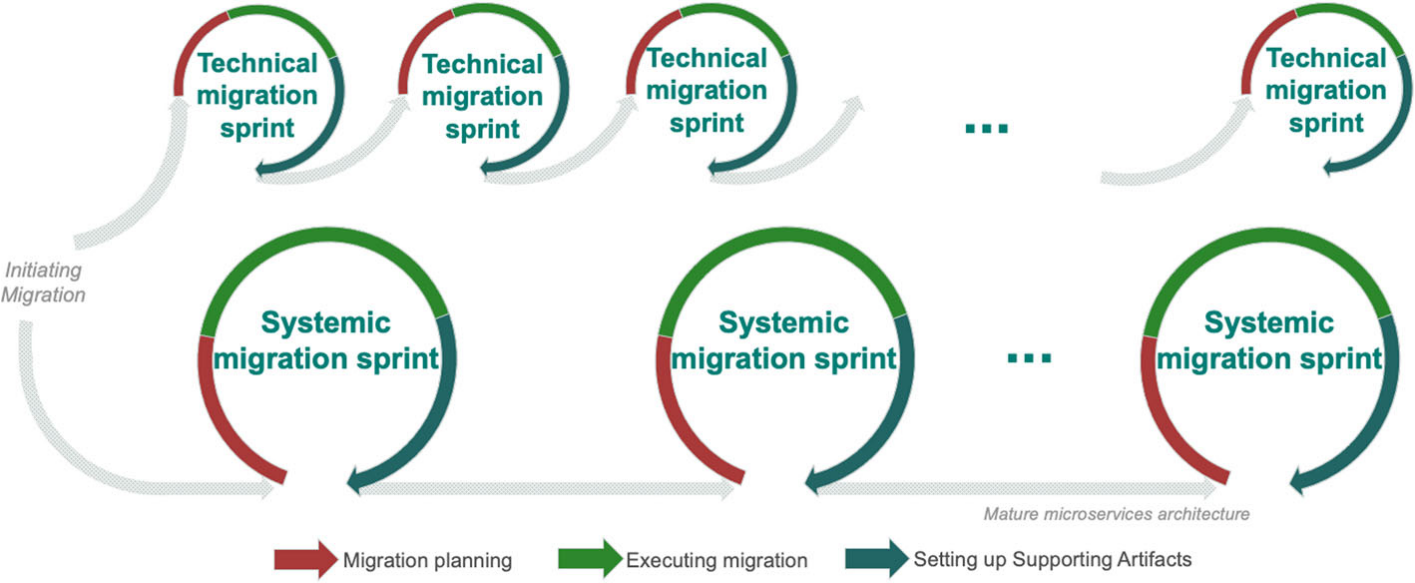
The iterative nature of the migration journey along the two different modes of change

As depicted in Fig. 2, microservices migration is an iterative process entailing two modes of change.

#### Modes of change

The identified modes of change are, as presented in Fig. 2, *1) the long-term journey*, which we refer to as the *systemic migration* and *2) the short-term journey*, referred to as the *technical migration*. The systemic migration is about the structural, organizational and business-oriented aspects of the migration. Additionally, the scope of the systemic migration is on the global software architecture transition that is required when an organization commits to a MSA migration. The technical migration is on a narrower scope, focusing on the technical realization of a migration towards MSA. Often the technical migration considers only the specific technical changes or one application on a subsystem. Specifically, it is about the evolution of a software system and changing its artifacts (e.g., concrete system architecture, monitoring solutions, etc.). Neither of the two modes of change should be understood as a one-time process. Instead, both modes of change are repeated in an iterative manner (in the case of the technical migration on the same or different technical systems), until the migration is completed. Note that this end point does not necessarily imply that all subsystems have been migrated. Based on our observations, many organizations will consider a migration completed even if some elements of the system remain in the old form.

The two modes of change typically happen in parallel (even though they are connected and influence each other). Each iteration of the systemic migration has a long-term scope and often contains multiple iterations of the technical migration, as will be illustrated in the example case below. For example, interviewees I1, I3, I5 and I6 mention that system designers and business analysts were inexperienced with microservices and needed some iterations to grasp the new concepts, during the first migration attempt. A common denominator across most investigated migration cases is that the migration project is more of an on-going and re-occurring project rather than a one-off execution of steps. On the one hand, a migration can be taking place in different parts of the system simultaneously on different teams. On the other hand, a migration also happens in increments or sprints from the same team, when evolving the same part of the system.

Both modes are different from each other concerning *1) the involved roles* and *2) their responsibilities*. During the systemic migration, roles such as senior engineers, system architects and managers are responsible for making decisions about the overall direction of the migration, the targeted architecture, and the tooling that will be used. On the other hand, the technical migration takes place in the daily work of engineers, who are responsible for changes closer to technical implementation.

#### Phases

We use three thematic phases for structuring both, the systemic and technical migration, as shown in Fig. 2. The phase of *Migration planning* is about planning and preparing for how the migration will be executed. The activities of this phase take place at the start of a (systemic or technical) iteration. The phase of *Executing the migration* is about modifying the software architecture (systemic migration) and source code (technical migration). Finally, the phase of *Setting up supporting artifacts* is about setting up additional infrastructure for the development and other technical artifacts required to support the microservices paradigm.

#### Illustrative example

In the following we use a running example, which is an imaginary construction of a case migration, based on our experiences from the interviews. We use the profile of an organization that has a system with a couple of decades in prior software development. This software was firstly developed to support the organization’s business, but evolved into a crucial value adding part of its core business. Multiple departments and teams are working in different parts of the software and the entire organization needs to synchronize for updating it. As the organization grows with more customers and more features, the software grows to the extend that it is difficult to manage, maintain and run with reliability. This imaginary organization considers the option of generally restructuring their software and specifically migrating towards microservices. The migration organization will follow an iterative approach, with several systemic change cycles that each of them contain several technical change cycles. The organization’s management, enterprise architects and senior software engineers investigate the overall systemic change that will be taking place on the long term. Software developers investigate how their software artifacts will change in a more immediate time frame.

### Systemic Migration

**Fig. 3:**
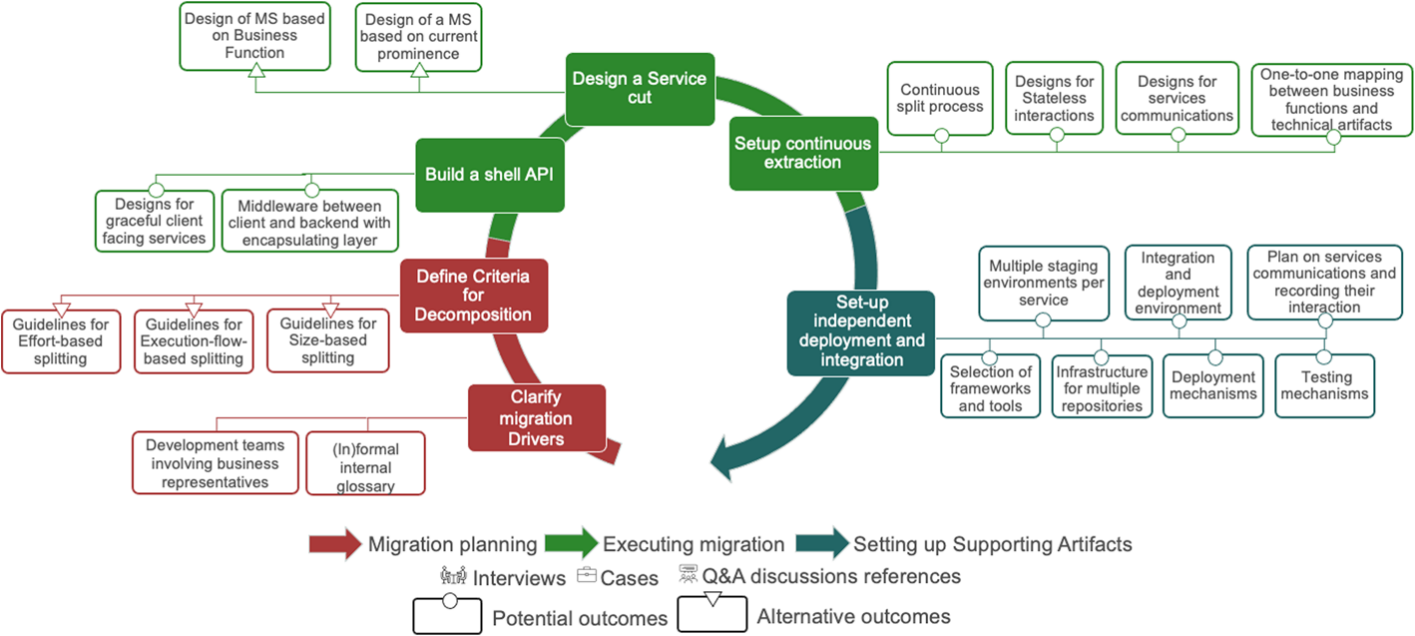
The seven activities in the Systemic Migration towards a MSA with their solution outcomes

The systemic migration has two activities in the phase of Migration planning, three in the Migration execution phase, and one in the phase of Setting up supporting artifacts, as shown in Fig. 3. Each activity has a set of solution outcomes that are described in detail subsequently.

#### Clarify the Migration Drivers

##### Context

The first identified activity in the studied migrations is to *clarify the migration drivers*, being business drivers or technical drivers. Clarifying the business and technical drivers is needed to align with all stakeholders on the requirements of the migration. In most migration cases we investigated in the interviews, there was a large process of deliberation in which different stakeholders, with different concerns and interests, had to exchange their views and align with each other. According to some highly experienced interviewees (I10, I13 and I17), this is the ideal phase to obtain a critical stance on microservices and consider if it is really a good fit for the overall objectives at hand. In fact, I17 described how missing this discussion at this stage made it costly later on to abort the migration and revert to a previous architecture once again. After all, and as I2 also discussed, MSA is not suitable for all types of systems.

*“It depends on the type of the product, the audience, time constraints. [If] I should build now a system for a small company, I would build something monolithic. There’s no serious reason to start building in microservices for that company.”* - I2

##### Addressed problems

- Engaging top management in the migration initiative to align with business benefits and ensure the budget of the migration, which is not always easy to get.
- Resolving doubts about whether microservices are a suitable architecture to achieve the organization’s goals.

##### Solution outcomes

**Fig. 4:**
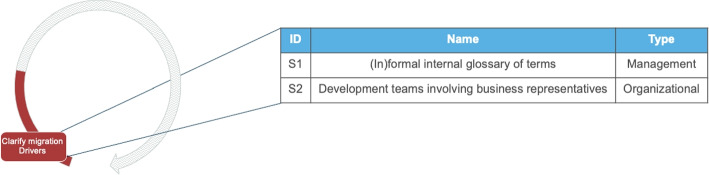
Possible solution outcomes for the activity “Clarify business and technical drivers”

Related to this activity, we identify two possible solution outcomes (see Fig. 4). The first (S1), is the design of an internal terminology to communicate effectively among people with different backgrounds. Microservices migrations often entail modernizing systems and, more often than not, require new synergies among people from different disciplines, who have not worked together before. The Domain-Driven-Design-based strategies that are prominent in microservices (Newman , 2015) require domain specialists. The interviews also revealed that business people and software engineers from different specializations need to work together on complex tasks. This multidisciplinary collaboration requires defining a common vocabulary. We characterize this solution outcome as management-oriented because it is about managing a smooth collaboration between people with different backgrounds.

The second solution outcome (S2) in this activity is a change of the structure of the development team, such that the business perspective is represented. A prominent way to do this is to include business representatives into the teams. We have observed multiple posts from engineers claiming that this is an essential element in order to achieve domain-driven-decomposition and business-centric design of the software architecture. Including a person who takes a business perspective, can enable a team to perform a decoupling of processes based on business logic, minimize synchronized responses and, therefore, allowing a more stateless design early in the migration. This solution outcome is characterized as organizational because it has to do with how teams are structured in the organization.

*“Not only do you need to ensure that the motivation behind replacing monoliths with microservices is sound, but also you need to step outside the monolith and revisit the actual business and begin partitioning that instead.”* - StackOverflow Discussion^3^

##### Execution challenges

- Successfully defining migration drivers requires alignment between business and technical teams. Finding this consensus is time-consuming and entails a learning curve.
- Estimating the returns on investments of a migration is essential to constructing the business case for the migration. However, such estimations naturally entail high uncertainty and guesswork.

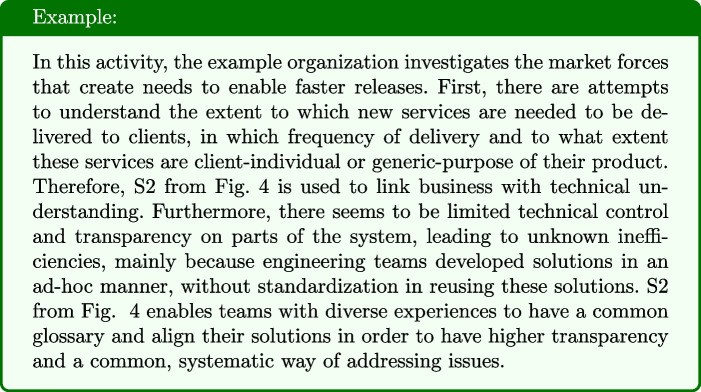

#### Define the Criteria for Decomposition

##### Context

Once alignment among core stakeholders is achieved comes the activity to *define the criteria for decomposition*. This step uses the input from stakeholders to define the goals of the migration and a service decomposition rationale. For example, both I5 and I8 mention how the migration is driven by two groups: management, which tries to market the company’s technology as modern, and the software development teams, who try to eliminate dependencies and bottlenecks in development. The objective of this activity is to estimate the (business) impact of deciding how to pursue the migration and translate the migration’s motivation into concrete guidelines. Common points of discussion in the data revolve around which parts of the system should be seen in isolation to accommodate (among others) business, regulatory and security requirements.

##### Addressed problems

- A lack of clear criteria for decomposition can lead to individual teams prioritizing their own needs at the expense of others’ needs. Criteria for decomposition can serve as a way to communicate to teams which non-functional properties are particularly important and should be prioritized.
- Existing Best Practices are often perceived as unclear, generic or challenging to follow Waseem et al. (2021). Hence, this activity is needed to interpret existing practices and adapt them to the specific company or project context.

##### Solution outcomes

**Fig. 5:**
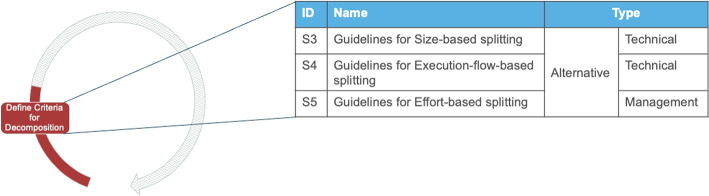
Possible solution outcomes for the activity “Define criteria for decomposition”

The solution outcomes in this activity relate to defining the principles in which the architecture will be split. In this activity three different solution outcomes emerged, which can be seen as alternative decomposition “rules of thumb”, that prioritize size, execution flow, or effort, as indicated in Fig. 5. Decomposing based on size (S3) entails defining an “ideal” size (measured, for instance, in the number of lines of code that a microservice should have), whereas decomposing based on execution flow (S4) is about using patterns of dependencies on the state of components to merge them into one microservice, or split them accordingly. Both S3 and S4 need a technical understanding of the system, to develop pragmatic guidelines. Finally, decomposing based on effort (S5) is visible when prioritizing microservices split outweighs the effort required to perform the split. Therefore, in the solution, engineers estimate the potential impact of the split and the effort required to achieve the impact and decide accordingly. This potential outcome requires input from management regarding the effort threshold that should determine whether it is worth to perform a split.

*“You definitely don’t want to implement complex, hardly maintainable logic for object creation just to be able to handle more requests than your system would ever have”* - StackOverflow Discussion^4^

##### Execution challenges

- Achieving consensus between different stakeholders in prioritizing different metrics is a challenging task. There are many constraints to take into consideration Gysel et al. (2016); Mazlami et al. (2017); Fritzsch et al. (2019), and likely no decision will make all stakeholders happy.

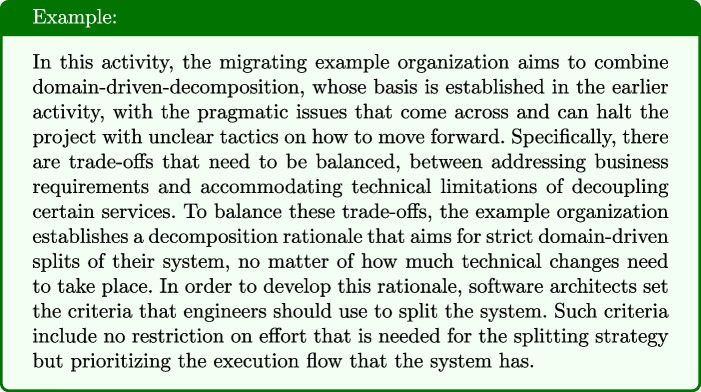


#### Build a Shell API

##### Context

Once migration requirements are clear and decomposition criteria are defined, most interviewees mentioned the need to manage the old system during the migration. This includes, for example, investigating how the (new) microservices will communicate with the legacy system during the transition phase. A way in which this takes place is by *building a shell API* around the old system. In some cases, microservice migration takes place by building an entirely new system from scratch (e.g., I1, I3, I6). However, even in these cases a shell API around the previous system’s data layer is required, enabling the usage of data from the old system. This activity is on the systemic migration because 1) it is about the overall structural change of the system and 2) influences the global architecture of the system that will emerge during the migration. The focus is on standardizing the communications between services.

##### Addressed problems

- Isolate (parts of) the system to maintain existing core behavior as the system architecture is refactored.
- Maintain a controlled starting point, but also controlled intermediary stages of the architecture until it reaches its microservices structure Knoche and Hasselbring (2018).

**Fig. 6:**
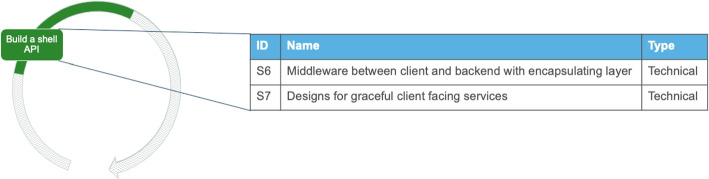
Possible solution outcomes for the activity “Build a Shell API”

##### Solution outcomes

For this activity, two solution outcomes emerged, as shown in Fig. 6, that both require technical input on the systemic change. The potential outcome (S6) of this activity is an encapsulating layer that maintains the behavior of the application, in order to achieve a seamless experience while changing. For example, I11 (among others) built a middleware that acted as an internal API around the legacy system, and did not use any data coming from any other place. However, this was only possible with dedicating development time in maintaining and evolving this middleware.

*“The first step was to take the backend as a whole, as one piece [...] connect it with a linked library that is imported in the UI, and then we built an API around it.”* - I11

Hence, building a shell API is creating a layer between client side and the backend data calls. This allows to maintain the behavior of the application the same and gradually change the way the backend is called.

*“Create a ‘collation layer’ that exposes queries to return global views for the client [...] The collation layer can protect the end client from such changes.”* - StackOverflow Discussion^5^

The second potential outcome (S7) is about ensuring that the behavior of the application is maintained the same while changing. This way, it is intended to to achieve a seamless experience to the clients while changing the software architecture that structures the system. Software architects in this stage, design and communicating requirements to engineers that all client facing functionality should change gracefully. Meaning that the change is not visible to the customers unintendedly.

*“Some of the services might go down because of disruption or deployment, in which case you have to think about handling these situations gracefully (for example apply circuit breaker”* - StackOverflow Discussion^6^

##### Execution challenges

- Building the shell API requires engineers with intimate knowledge of the original system’s behavior to lead the initiative. These engineers need to be given sufficient time to develop a reliable API that retains the original behavior as precisely as possible.

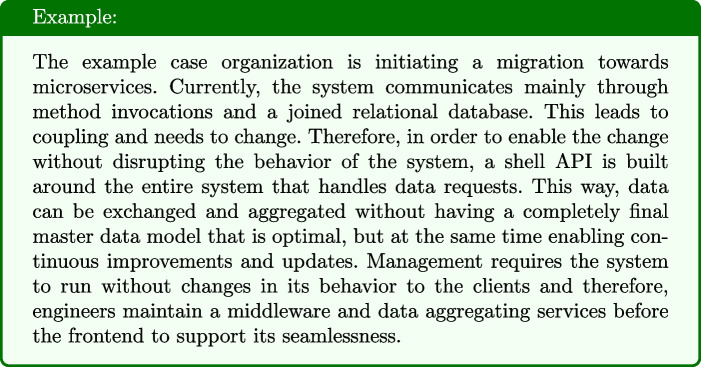

#### Design a Service Cut

##### Context

**Fig. 7:**
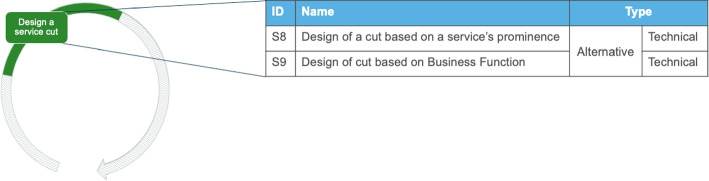
Possible solution outcomes for the activity “Design a service cut”

As shown in Fig. 7 the first decomposition takes place by *designing a service cut* for extracting one service or performing a first split, sometimes in combination with developing a new microservice. The activity is about designing the architectural split of the system and drafting the future architecture. Also, it includes the documentation of the first split of the system, in order to use it as a Proof-of-Concept or template in future splits and during the technical migration. In this activity we find evidence for two alternative ways that can be executed and, typically, only one of them is followed. This can result in a hybrid architecture with a small monolith within the MSA, according to I2 and I11. It is worth noting that a hybrid architecture does not necessarily appear when a new architecture is developed in parallel to the existing one. A trade-off of this activity is balancing upfront investment with the need for an immediate design split.

##### Addressed problems

- Decide which parts of the system to start decomposing.
- Scope the discussions to a realistic level of granularity, which can be achieved in the current stage of the migration.

##### Solution outcomes

Designing a service cut can lead to two alternative solution outcomes. Here, the guidelines selected in activity “Define the criteria for decomposition” are used. The first alternative solution outcome (S8) is about designing the extraction of one service on the basis of a prominent system part. For example, the extraction can take place on a big part of the system or on a commonly used part. For example, when extracting a large, cohesive part of the existing system as a microservice. However, we noticed that the question, what a large part is, is relative to the current size of the existing software. The designed microservice can be subject to further cuts in future iterations. The alternative (S9) is about designing the extraction based on a clearly defined business function. Hence, the extraction takes place solely based on business requirements on the responsibilities of the services. Engineers mentioned that they make service cuts from the frontend until the database to isolate a feature that should be provided as a service. These vertical cuts are designed with either of the two approaches (S8 or S9) and used in the technical migration to guide the technical split.

*“Microservices should be more of a vertical partitioning of your application and not a horizontal one. In my opinion it’s better to think in terms of business function partitioning rather than “converting” an existing monolith.”* - StackOverflow Discussion^7^

##### Execution challenges

- Decide on the starting point for the decomposition is inherently challenging. Starting with core functionality is often difficult due to a plethora of dependencies, but adds most direct value. Starting from more peripheral functionality requires less effort but is also perceived as less useful.

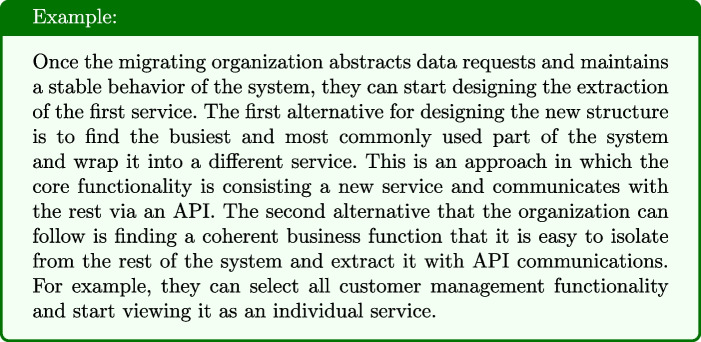

#### Setup Continuous Extraction

##### Context

Senior engineers and software architects use their newly acquired knowledge from the first split and then typically define the granularity of the services and *repeat the extraction*. This activity is about setting up a continuous extraction process that can be executed by engineers in subsequent splits of microservices, for example in the technical migration. Therefore, it includes defining the granularity and planning the re-extraction of services.

*“is a continuous problem defining how big the area of concern is [...] I have some functionalities and are these one service, multiple services or something to be added in an existing service?”* - I15

**Fig. 8:**
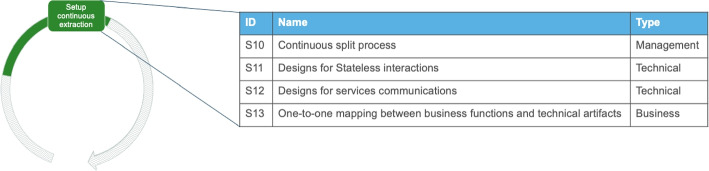
Solution outcomes for the activity “Setup continuous extraction”

##### Addressed problems

- Propagating the migration to the entire organization, with a systematic way of making technical decisions (Waseem et al. 2021).
- Propagating the new software development mindset to the organization, via setting up a process that individual development teams who are not experienced with microservices can follow throughout the organization.

##### Solution outcomes

As shown in Fig. 8 the first solution outcome (S10) is a continuous split process that can be given to engineers (in the technical migration). The process helps them to accelerate the extraction of service, when executing the decomposition of different parts of the system that are similar. Four Stack Overflow posts support the need to standardize and organize tasks that have to do with decoupling the software, as migration projects evolve. Consequently, we characterize this outcome as management-related.

The solution outcome S11 seems popular among engineers that are in more advanced migrations, and it is about developing refined execution flows of services that eliminate ’statefulness’ as much as possible. Re-designing parts of the systems with the mindset/objective of eliminating stateful interactions between its constituent elements can lead to creating more independent services and therefore, reduce coupling (i.e. direct connections that bypass endpoints or waiting for chained services). The solution outcome S12 is about designing data aggregating services to start abstracting communication to an architectural level rather than a program level. Data aggregating services need to be designed for facilitating the APIs developed in earlier activities. These services are responsible for gradually taking over the communication between services in a structured and controlled manner. Both S11 and S12 need input from technically astute senior engineers.

Finally, S13 is a one-to-one mapping between business functions and technical artifacts. The emergent idea of this solution is to isolate the scope of technical artifacts to a clearly defined unit of business and limit the scope of a business unit to one artifact. The objective is to develop the self-containment of services and help in reducing their coupling as well as the achievement of solution S11.

*“The ideal Self-Contained System would be completely independent of other Systems, would cover one or more highly cohesive business functions (in full depth from the UI to Persistence!), and would be not calling any other system synchronously.”* - StackOverflow Discussion^8^

##### Execution challenges

- Changing the software development mindset of engineers requires time. In addition, the coordination of all involved stakeholders is not trivial. Particularly, having technical knowledge is necessary but not sufficient. Knowledge of the microservices-based software development paradigm needs to be conveyed to engineers effectively so that they can start performing the migration in different parts of the system.

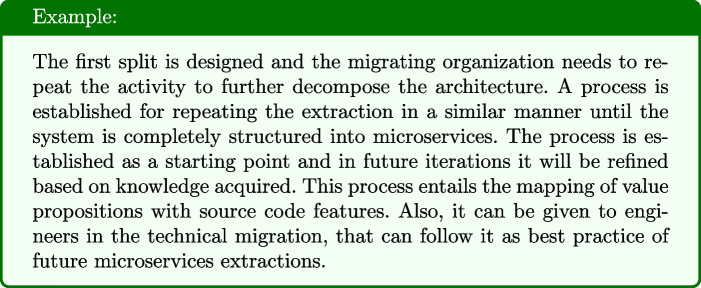

#### Setup Independent Deployment and Integration

##### Context

Increasing the number of microservices results in a need for setting up supporting artifacts for new development, integration and deployment issues that are surfacing with the adoption of microservices. Such artifacts are deployment environments, testing processes and logging or monitoring. This activity is about *designing and configuring the infrastructure for integrating and delivering microservices individually*. The activity needs to accommodate a distributed system with independently developed sub-systems. The scope is the software release architecture that is going to be used in the technical migration. This scope requires prior experience in introducing a new tool chain and changing the current status quo of development. Hence, this activity is also about deciding the detailed elements of the automated release architecture that the technical migration will use to independently deploy and test services. Consequently, this leads organizations to *facilitating independent and dynamic testing and deployment* for rolling-out new versions of the application.

##### Addressed problems

- This activity, helps to avoid an un-standardized delivery by establishing the central infrastructure that supports integration and deployment of multiple individually developed and loosely coupled, autonomous services. Setting up independent deployment and integration, establishes a unified model for running the software and for its composition to the complete system.
- In addition, this activity intends to unburden developers from the required configurations to integrate their services to the entire system.

##### Solution outcomes

**Fig. 9:**
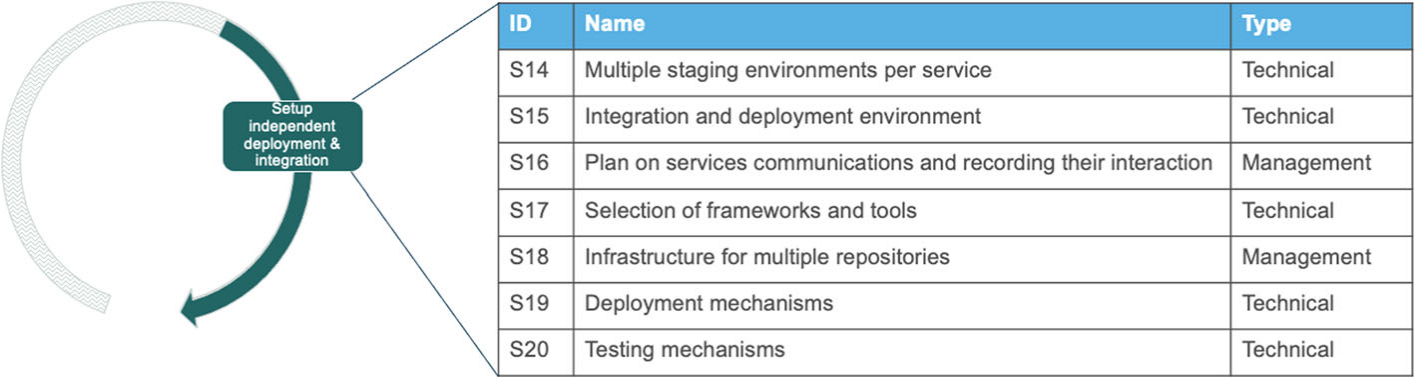
Possible solution outcomes for the activity “Setup infrastructure for integration”

We identified the solution potential outcome S14, as shown in Fig. 9 which is about planning for configuring in each individual service the infrastructure for coordinated integration and deployment with the rest of the system. The outcome is a standard way of having these environments in order to achieve a coordinated and consistent way of releasing different microservices into one system. This outcome paves the way for developing capabilities that enable the seamless merging of multiple deployment environments of sub-systems. For example, with setting up CI and CD pipelines and writing scripts that will make the integration happen in a systematic way. These capabilities enables to integrate different individual services, in different versions with each other.

*“We do weekly release cycles. Each service/component located in the separate git repository. ’A release’ - is several features that we put into wild. Usually only several components should be updated.”* - StackOverflow Discussion^9^

The second solution outcome (S15) is about configuring the part of the infrastructure in which the different individually developed services (e.g., in a release train) get integrated. A solution that appeared in this activity is regarding the preparation of integration and deployment environments. This includes the configuration of development, testing and production environments and mechanisms to manage the quick release and revert from different versions of services to ensure compatibility. The solution here is basically the resulting DevOps architecture for releasing software. This solution requires a technical skillset of setting up such automatic environments that control releases. This includes setting up staging environments and processes, dynamic testing and delivery processes. Such processes include both automated structures but also more manual activities such as setting the communication among services.

*“we have some automated testing [...], when everything is OK, the service is deployed on a staging Kubernetes cluster to be tested, and if everything is OK, it is promoted to production.”* - I3

In S16 we identify a solution outcome about planning how to record interactions between services. Logging is perceived to be more crucial in the context of microservices than for other architectures, as a lack of it leads to often unexplained system behaviors. For example, both, I1 and I2, described how their systems behaved unpredictably and had unknown bugs surfacing frequently, in initial migration attempts. In these attempts, a proportion of the code was reused without adding any logging. Hence, the systems became non-transparent making them hard to maintain. The outcome is about facilitating cross-service communication through managing the execution complexity and altering execution flows in order to achieve asynchronous communication between services (as described by the answers to Post #73).

The identified solution outcome S17, shown in Fig. 9, is a required design where engineers with a portfolio of experiences in different types of tools select the most appropriate ones for their case. The focus is mainly on identifying frameworks and tools that will enable independent testing and deployment. This includes the selection of frameworks (in connection to the technical migration) that are more suitable for independent testing like Angular libraries or tools that will help in managing independent deployment like Azure’s Service Fabric or hosting Lambda functions in different CloudFronts to achieve high availability across different time-zones. The management-oriented outcome in this activity is S18 which is also a required design. S18 is about setting up the general infrastructure changes that are needed for individual repositories (to be used in technical migration). Its appearance is quite prominent in StackOverflow discussions and it is about preparing multiple repositories for individual services. This allows the independent deployment but also requires setting continuous integration pipelines and keeping track of costs that might arise.

*“We where putting each micro-service in a separate repository as the Jenkins pipeline was build in a generic way to build them that way[...] This was helping us in some cases. In general you should also consider the cost.”* - StackOverflow Discussion^10^

The solution outcome S19 is about introducing deployment mechanisms, including the ways that applications are run in different virtual machines and the development of circuit breaking to manage downtime without influencing the entire system. Finally, the solution S20 is regarding seting up testing mechanisms, including the different ways that engineers use for testing containerized code, software behavior with load balancing and integration (e.g., using Consumer-Driven-Contract testing). An interesting approach on testing in S10 included the need to mock all cross-microservices calls to allow the execution of unit and integration tests.

*“For testing purposes you should mock all cross micro-service calls and not block your Unit/Integration test by other services. This is a common approach in micro-services systems. This way your tests will not depend on other micro-service.”* - StackOverflow Discussion^11^

##### Execution challenges

- Setting up independent deployment and integration entails the (often complicated) configuration of infrastructure solutions Waseem et al. (2021). This infrastructure does not in itself add functionality to the product. Therefore, stakeholders might not perceive the activity as directly value-adding.

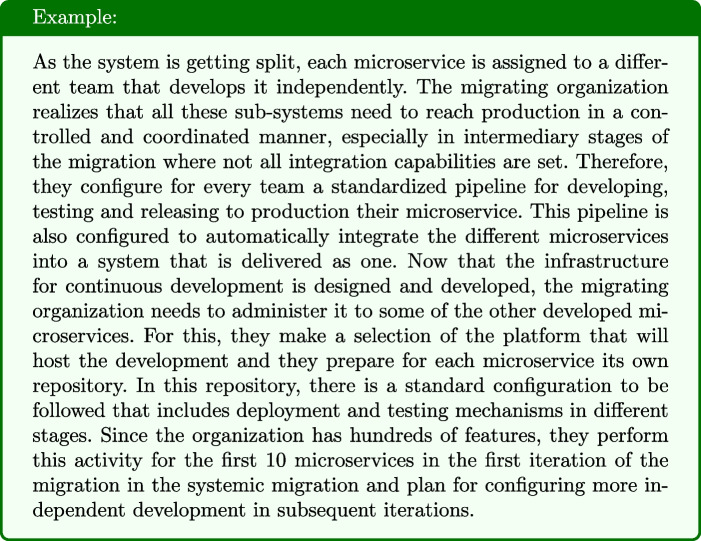

### Technical Migration

**Fig. 10:**
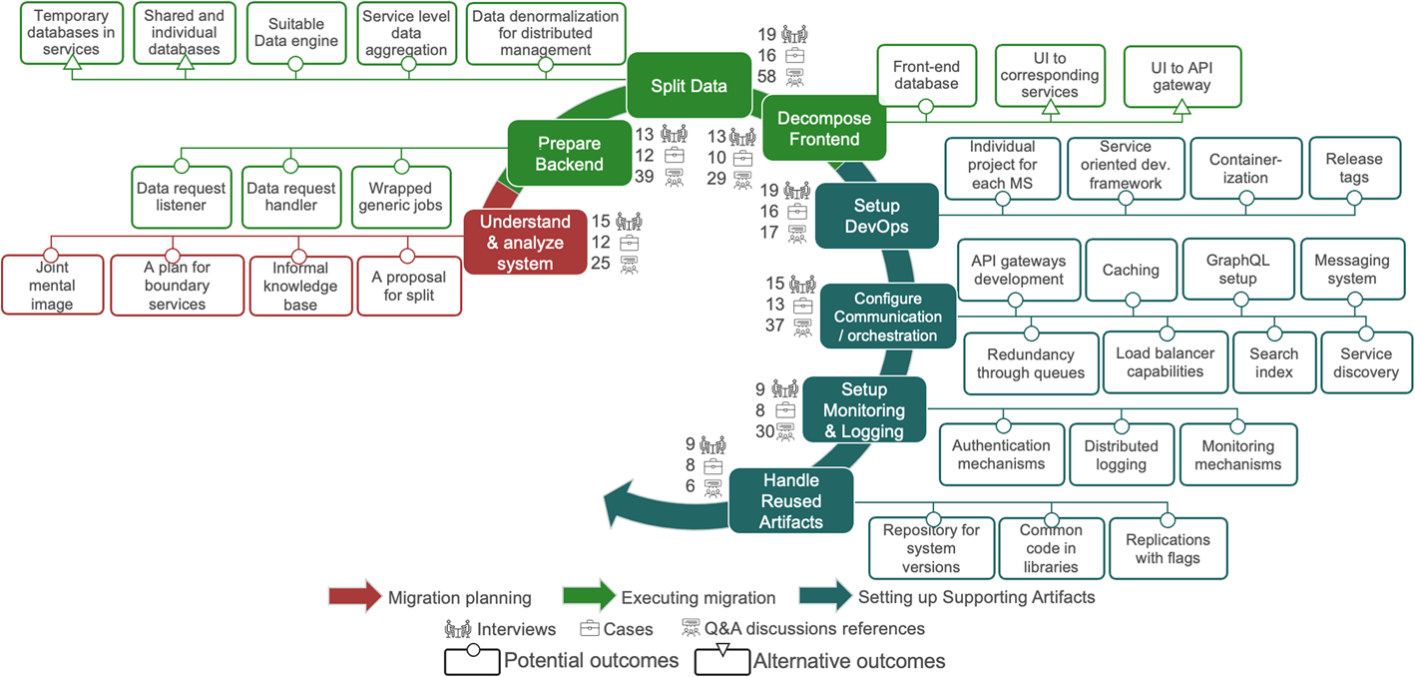
The nine activities in the technical migration to microservices

On the second level of microservice migration, the technical migration, we dive into how the software itself changes in time, as shown in Fig. 10. Technical migration iterations are more short-running – typically, multiple iterations of technical migration are repeated during one complete iteration of the systemic migration.

Our analysis reveals that activities in the systemic migration are on a too high level of abstraction for technically realizing the migration. Thus, they need to be complemented for defining the specific technical activities that will implement the change on a technical level. The activities on the technical migration operationalize those of the systemic migration. The technical migration has one activity in the phase of Planning, three in the phase of Executing and five in the phase of setting up supporting artifacts.

#### Understand and Analyze System

##### Context

First, development teams start from *understanding and analyzing the system*, which is the contextual information of the application and its purpose. This activity is on the technical migration because it is about understanding in detail the specific parts of the system and planning for the technical changes that will take place. In addition, in this activity we list outcomes that are specific about microservices and have to do with migrating an existing system that is already developed. The analysis of the interviews did not reveal many details on this activity. However, posts from StackOverflow often described this problem extensively. These details are presented in the form of four solution outcomes.

##### Addressed problems

- The activity helps engineers to maintain a perspective in which the technical constraints and goals of the technology are not the dominating factors, but rather the means to achieve business outcomes.

##### Solution outcomes

**Fig. 11:**
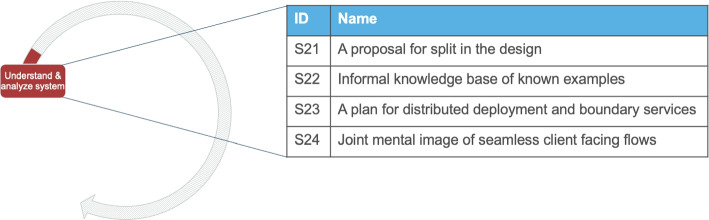
Solution outcomes for the activity “Understand and analyze system”

The identified solution outcomes, presented in Fig. 11, cover both business, technical and management aspects of a technical migration iteration. The first outcome (S21) is a design split, which is referred to in 20 Q &A discussions. The designed split is derived from analyzing the domain, for example based on a master data model, execution flows, and usage metrics of specific parts of the system. This way, engineers gain a data-driven understanding of the system’s domains. Additionally, engineers often need to estimate the impact of the potential number of connections per microservice to estimate the complexity and overhead in development and maintenance. This solution outcome uses information from the activity *Define criteria for decomposition*. Also, S21 uses the *designed service cuts* from the systemic migration and details them with the complete set of technical artifacts that will be altered.

The second outcome (S22 in Fig. 11) is about forming an informal knowledge base about existing practices and experiences from other migrations. This allows engineers to analyzing in depth the business logic and see parallels to other projects. Another solution outcome (S23 in Fig. 11) is a plan for defining the boundaries of the split services and their deployment. This outcome is about planning for distributed deployment and development of boundary services. The focus is on mapping the existing business logic to the new deployment paradigm and preparing the organization for it. For example, anticipating and planning task-based user interfaces that are developed separately and integrated together. The last solution outcome (S24 in Fig. 11) of the activity is (based on the *internal glossary of terms (S1)*) establishing a common view on the required seamless client facing flows.

*“If the customer quickly hits refresh - he expects to see his new name, as we don’t want to explain to the customers what’s eventual consistency”* - StackOverflow Discussion^12^

##### Execution challenges

- Migration initiatives are often fragmented, taking place when there is some time left, next to the development of new features. Thus, it might be challenging to maintain a momentum in the change. Therefore, in this activity, involved stakeholders need to *1)* take into account the required flexibility of the migration project, and *2)* prioritize what parts of the system need to be re-engineered first and what can wait for future iterations of the change. Finally, unclear communication and knowledge sharing can make the migration dependent on some individual engineers.

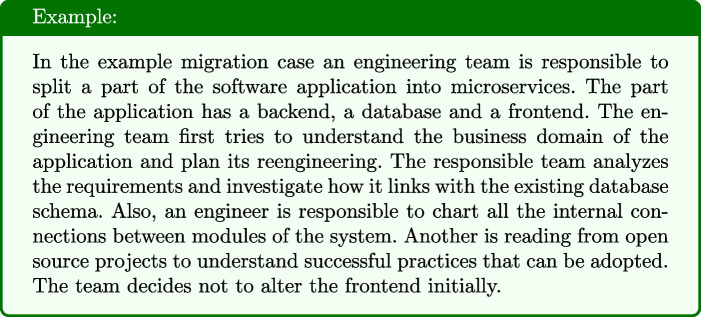


#### Prepare the Backend for Decomposition

##### Context

In the next activity, teams need to *prepare the backend for decomposition*. According to the interviews analyzed, we observe that the backend is often the starting point of the migration. This is in line with engineers’ discussions, seemingly due to the already organized nature of the backend and middleware. This activity takes the designs developed in the systemic migration and translates them into technical change in the system (being designs for a shell API or a service cut for example).

##### Addressed problems

- Define the often unclear functional area of the software to start decomposing different services. In preparing the backend, the software can start getting decomposed and organizing its structure for making it less coupled and more controlled.

**Fig. 12:**
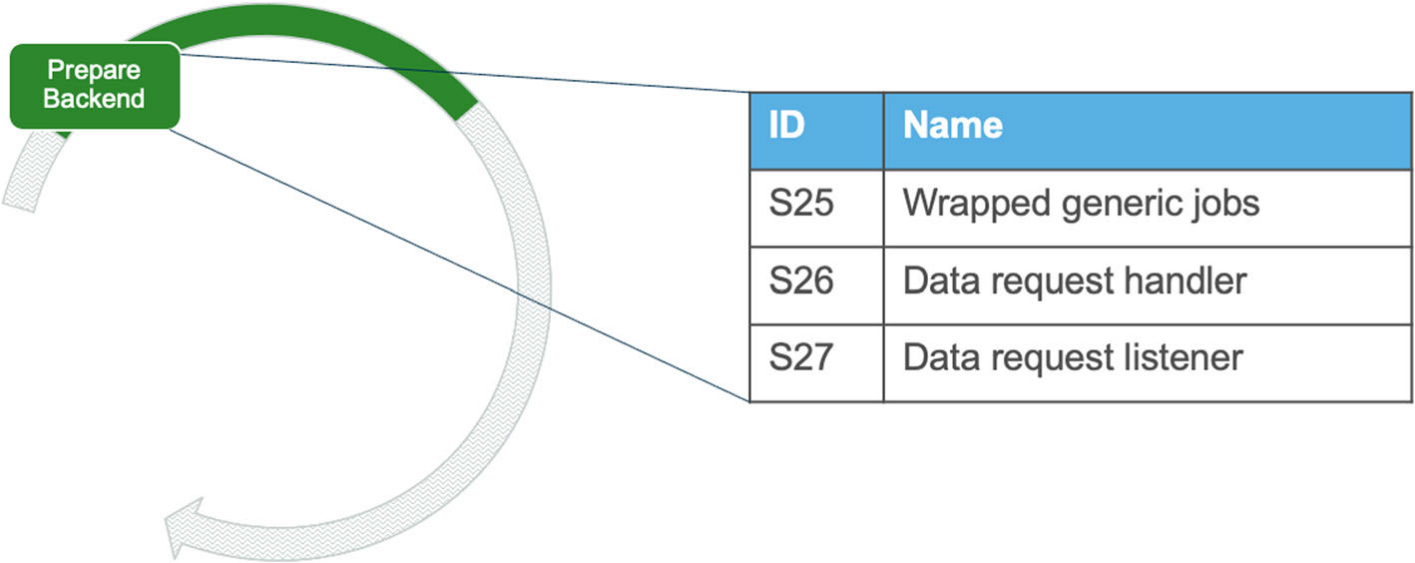
Solution outcomes for the activity “Prepare the backend for decomposition”

##### Solution outcomes

The solutions to the activity for preparing the backend are related to the way that the application is getting structured and they are among the most discussed solutions in StackOverflow.

The first potential outcome (S25 in Fig. 12) identified regarding the backend is the wrapping of generic jobs of the application. Specifically, engineers try to separate functionalities that are commonly implemented and used in the application, in order to expose them for reuse. These functionalities can be based on usage from the rest of the system, as well as basic functionalities that are in required in multiple places (e.g. authentication). In this solution, the boundaries of services start to get defined in the backend and more extensive structuring starts to take place.

The solution outcome S26 in Fig. 12 is preparing designs and implementation with the architectural style of having a data request handler (e.g. in the form of APIs) that manage the communications between services. A data request handler as derived from the analysis is basically the way of facilitating communication between services via a single entity that manages all data requests and distributes responses to the requesting service. The predominant ways of implementing such an entity is via a (central) service that manages all data requests from adjacent services and aggregates their data, or an API gateway that refers to the requested data sources with a unique address and manages the data aggregation. This solution has the benefits of a more organized structure and being easier to manage, since data aggregation takes place in a single place. On the other hand this also results in having a central point of failure in the application and also a potential bottleneck that might compromise performance in certain cases.

Another solution outcome (S27 in Fig. 12) is preparing designs and implementation that follow an event-driven style and have a distributed implementation across services on how to exchange data with each other. The design of having data request listeners is facilitating a more direct communication between services via event-driven techniques in which services call each other directly. This approach can have the benefits of stronger performance if developed correctly. However, it can result in a less structured approach, that is easier to lose track of in large systems. Losing track of connections between services can lead in coupling through synchronized calls in certain cases. Since both designs have their own merits, many engineers mention that often a combination of the two is ideal.

*“In microservices each micro-service can talk to another base on two different communication style:Sync (REST is suggested) or Async (via message brokers)”* - StackOverflow Discussion^13^

##### Execution challenges

- Taking decisions on how to split certain parts of the system can be complex, considering that coupling might be quite high, source code might be unclear and, in many cases, the original developers are not available to answer questions about the system anymore.
- Preparing the backend may be revisited and re-designed as the engineering teams learn new solutions and set-up new infrastructure (e.g. in subsequent iterations). Therefore, engineers often mentioned the critical challenge of maintaining the flexibility required to update and extend the software’s structure later on.

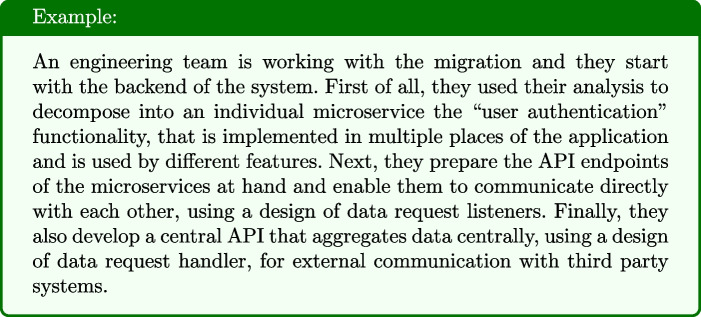

#### Split the Data and Data Migration

##### Context

Preparing the backend is often followed or takes place in parallel with *splitting the data* and *data migration*. This activity entails the reorganization of the entity relationships between data and also splitting the data into multiple databases. Also, since relational databases are fundamentally structured around coupling, many interviewees (e.g., I1, I2, I11) described how such databases are not always a good fit for microservices.

##### Addressed problems

- As part of the microservices migration, coupling in the data layer often has to be reduced or eliminated, while at the same time maintaining data integrity Wu et al. (2022). This activity initiates the discussion about removing coupling through service-level data aggregation as well as selecting the right data engine.

##### Solution outcomes

**Fig. 13:**
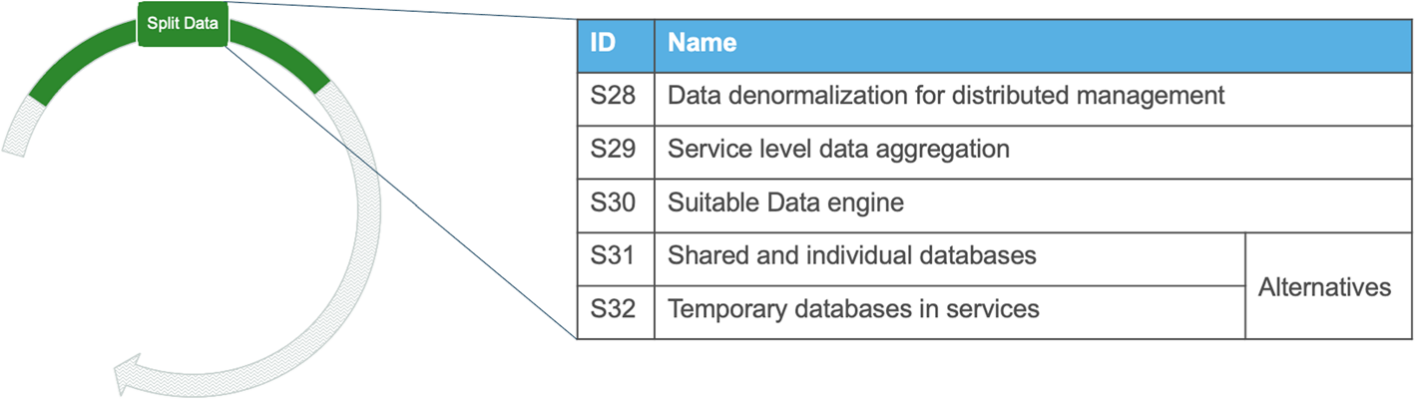
Solution outcomes for the activity “split the data and data migration”

As shown in Fig. 13, five solution outcomes are identified in this activity from which two are required designs, one is an solution outcome and two are alternative from each other. S28 in Fig. 13 is about denormalizing data that are going to be stored and structured differently in the new distributed architecture. This is a mandatory outcome because the data management layer will change at least on the ways that interacts with the application. Specifically, this solution is about splitting the database schema into independent data sources, that are separated from each other, suitable to feed individual services. This solution is what establishes the separation of data in order to enable service-level data aggregation (S29).

*“Given a database that consists entirely of related tables, how does one denormalize this into smaller fragments (groups of tables) so that the fragments can be controlled by separate microservices?”* - StackOverflow Discussion^14^

S29 in Fig. 13 is about the design and development of service level data aggregations and moving data aggregation from coupling on the data management layer, to controlled/regulated aggregation on the service layer. Specifically, this potential outcome is derived from 30 post references in StackOverflow. A natural consequence of data decoupling is that aggregation of data that would normally happen with simple ‘JOIN’ functions now needs to take place on a higher level of abstraction (the service level). Aggregating data on the service levels is a paradigm of data management that is different from the fundamentals of “databases-thinking” that most engineers learn. Therefore, it is sometimes perceived as a radical change for engineers.

Furthermore, S30 is aiming for an overhaul database engines upgrade towards modern ways of data storage. Specifically, since data are managed in a distributed manner, there is more flexibility in how different parts of the data are stored. Also, often the existing database engines are no longer suitable for the smaller-scoped services, the possibility (or need) to perform data-joins on a service level and the potential structure of data. Via changing the database engine, it is easier to initiate the often required data-schema refactoring process for decoupling.

Finally, S31 and S32 are alternatives to each other, and they are about either maintaining a shared database along with individual ones or maintaining temporary, often read-only databases in different services. S31 is having shared and individual databases. Specifically, engineers in their discussions mention that for microservices that have very frequent and performance critical data exchange, or large volumes of data exchange, it is sometimes virtually impossible to have separate databases and therefore, one could consider having shared databases with extreme caution. In addition, there is the need of estimating the potential costs of having data duplicates and compare with other alternatives, which is relevant for the business for budgeting the migration project. S32 is having temporary (read-only) databases of other microservices. This means that a microservice also stores data from downstream microservices in order to unburden the software from unnecessary requests. A request is considered unnecessary if it asks for mostly stable data that does not change often. This approach of database replicas is often described as a preferred compromise, compared to allowing coupling between services.

*“It’s not a bad practice to keep duplicated data of other services if you are going to achieve loose coupling.”* - StackOverflow Discussion^15^

##### Execution challenges

- Introducing and developing databases that are tolerant to failing microservices is quite challenging, but also crucial for maintaining data consistency and predictable behavior. This challenge is well known in literature and practice Wu et al. (2022), but is unavoidable in the transition to the stateless design that microservices entail.
- Data denormalization, particularly in legacy systems with complicated relationships between data entities, takes time, effort, and expertise. Furthermore, technical debt in existing database models, such as redundancies, misplacement, inconsistencies and varied formatting of data, can make data migration even more challenging.

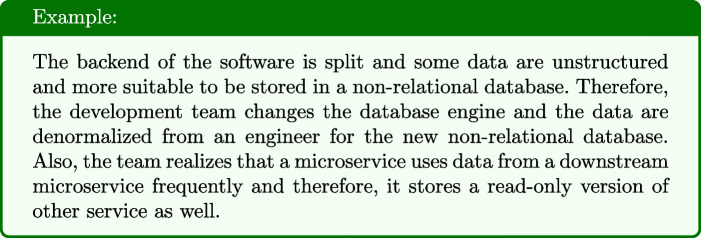

#### Decompose the Frontend

##### Context

Once the backend and data layer are split, at least partially, organizations can start *decomposing the frontend*. The frontend comes later, since ususally its decomposition depends on the other parts of the application. Hence, the frontend is often detached first from the rest of the application and only at a later stage (potentially) decomposed.

*“The frontend didn’t get split (yet). It’s doing one thing and there is high customization without it being overwhelming”* - I11

##### Addressed problems

- The frontend part of applications is often subject to particularly high coupling, often due to having highly standardized visuals across different interactions with a system. In addition, the design and development of a decoupled frontend has to consider both the interactions between individual services, but also interactions with the backend.

##### Solution outcomes

**Fig. 14:**
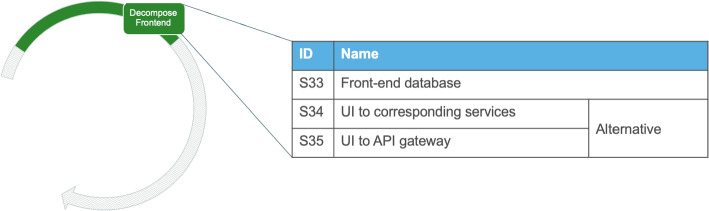
Solution outcomes for the activity “decompose the frontend”

We extracted a set of solution outcomes that engineers adopt in this activity and they are presented in Fig. 14. The identified potential outcome S33 is that of maintaining a frontend data-model. In this solution a replica is made of the needed data from services for direct usage in the frontend. This is a very similar approach to maintaining a temporary database in a service (S32) or a cache (S42). The solution outcome S34 in Fig. 14 is about structuring the front end by allowing every piece of the front end to communicate directly to a corresponding service for getting data and aggregating the view in the User Interface. This enables more flexibility in a seamless experience for users, but comes with more complexity in implementing, and often with an overhaul update of the frontend’s code. In contrast, S35 in Fig. 14 is a User Interface structure with a combined API that enables a single point of maintaining the frontend.

##### Execution challenges

- Frontends are customer facing and therefore, highly critical. Altering the part of the system that interfaces with customers needs to happen in a seamless manner to avoid service disruption.
- The code structure of frontends inherently entails the use of shared libraries and tight coupling in the used script-based frameworks. The unique and intertwined structure of frontend software requires more effort and time to decompose, which might not be viable considering that technologies used and customer interaction design might anyway change in time.

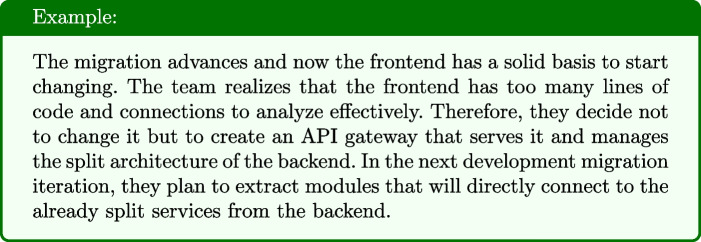

#### Set up DevOps Capabilities

##### Context

Many organizations indicated that when microservices are decomposed and the distributed system grows, it needs to be managed considering all the new attributes of the introduced architecture. Hence, there is typically an alteration in *DevOps* practices. This includes the structures that organize development and testing of microservices. *Testing mechanisms* are altered to support the distributed nature of the new architecture. Specifically, testing now takes place on different levels, starting from simple unit tests, to integration tests and to deployment tests. This activity materializes practically the designs developed in the activities of setting up integration, testing and deployment from the systemic migration.

*“testing specific combinations of microservices is super hard to achieve [...] I know that we are doing a lot of manual work, individual tricky solutions and hacks to make our test frameworks do what we want to test this complexity.”* - I18

##### Addressed problems

- Setting up DevOps capabilities is the activity that helps manage the changing infrastructure of the system. The ways that software is stored, organized, and even deployed are changing and this activity helps to introduce and maintain the required elements for running and managing microservices. This activity addresses the need to allocate the required effort and engineering resources to setting up the required capabilities for the organization’s DevOps. In addition, the activity provides the basic starting point for the required infrastructure to be in place.

##### Solution outcomes

**Fig. 15:**
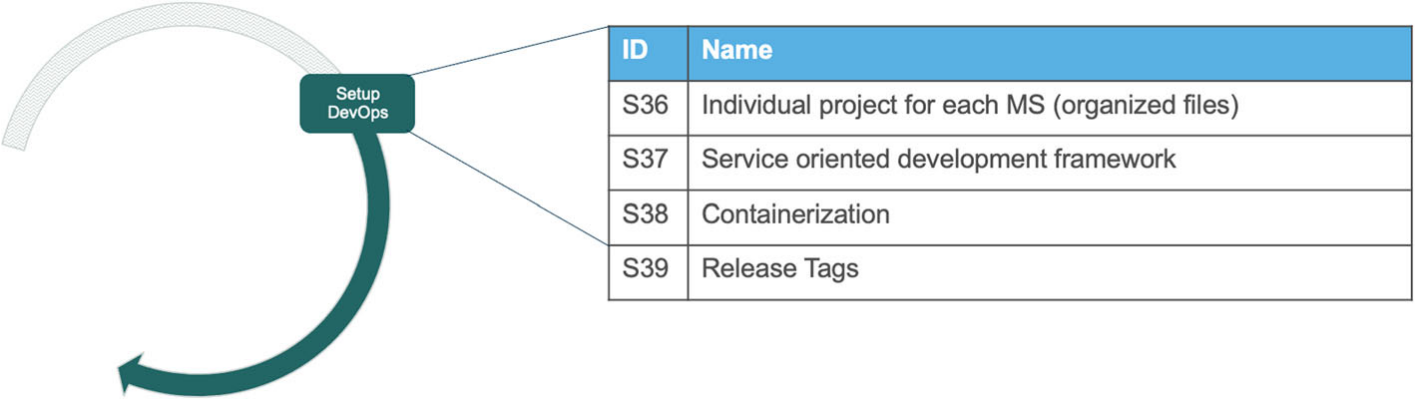
Solution outcomes for the activity “setup DevOps capabilities”

Mainly based on discussions from StackOverflow, we identified S36 in Fig. 15 which is, organizing and structuring each microservices’ files into their individual projects. In this, engineers have to create one individual project directory for each microservice (especially in cases that the existing system is stored in one big project). Worth noting, is that in migrations, it is typically important and effortfull to organize files rigorously, even if monorepo approaches are used. Hence, this solution outcome is a required design, mandatory to migrations. This includes the organization of files that are used by the application (e.g. stored in the cloud) or the files that compose the system (e.g. source code, individual libraries etc.).

*“So yes, ideally your each micro-service is a separate Django project. The best way to break this, first list down all the possible modules in your site or app.”* - StackOverflow Discussion^16^

The potential outcome S37 in Fig. 15 is identified jointly from online discussions and interviews and it is about having a service oriented development framework. For example, this includes a difference in development in which engineers are no longer required to have a local version of the entire application on which they develop. Instead, small parts of the software - microservices - can be developed separately and then get deployed in a staging server, following a predefined process. This introduces some new practices, including the development and management of APIs and independent services.

A popular solution outcome (S38 in Fig. 15) of transitioning towards microservices is containerizing different parts of the application. Posts referring to this included the way of encapsulating different parts of the system into a container, but also the organization of development and deployment of these containers into microservices. Therefore, quite naturally this solution includes the orchestration of containers in more advanced migrations using tools like Kubernetes.

Another solution outcome (S39 in Fig. 15) identified in this activity is regarding the integration and deployment of multiple independent services that are asynchronously developed and have different levels of maturity in their development. Therefore, release tags can be used to characterize components in production and clarify updates on new versions of the system.

*“You can also save some time with automatic git-tagging script for all your deployable components. The script would check if anything has changed in the master since the last tag, and, if it has, it would stick a new git tag on the repo.”* - StackOverflow Discussion^17^

##### Execution challenges

- Setting up DevOps requires specialized expertise and knowledge of DevOps concepts Waseem et al. (2020). As many interviewees mention, teams often lack previous expertise and experience on DevOps when they start migrating. In addition, configuring the required infrastructure inherently entails multiple governance challenges Wu et al. (2022).

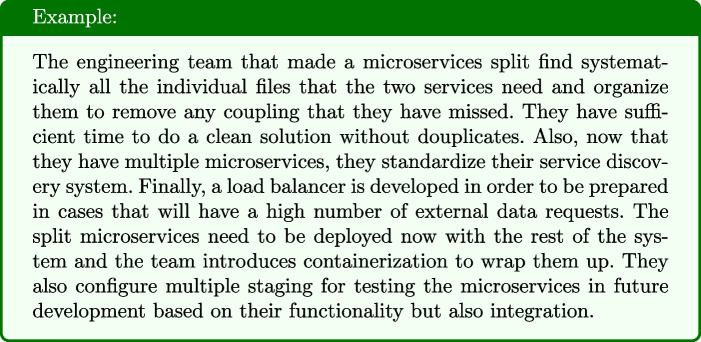

#### Configure Communication and Orchestration Capabilities

##### Context

Moreover, there are additional technical artifacts that introduce an overhead in development, that are needed to support the new architecture. Once the application is decomposed, teams come across the need of developing *Communication and orchestration* between services. Many teams mention how a microservice-based architecture propagates the complexity of different pieces of software to the communication layer. Therefore, the way that microservices communicate with each other needs to be carefully designed.

##### Addressed problems

- Microservices that are developed and run independently need to communicate and coordinate to compose the complete system. There are many ways to facilitate their communication and this activity initiates the configuration of the required tooling for communication and service orchestration.

##### Solution outcomes

**Fig. 16:**
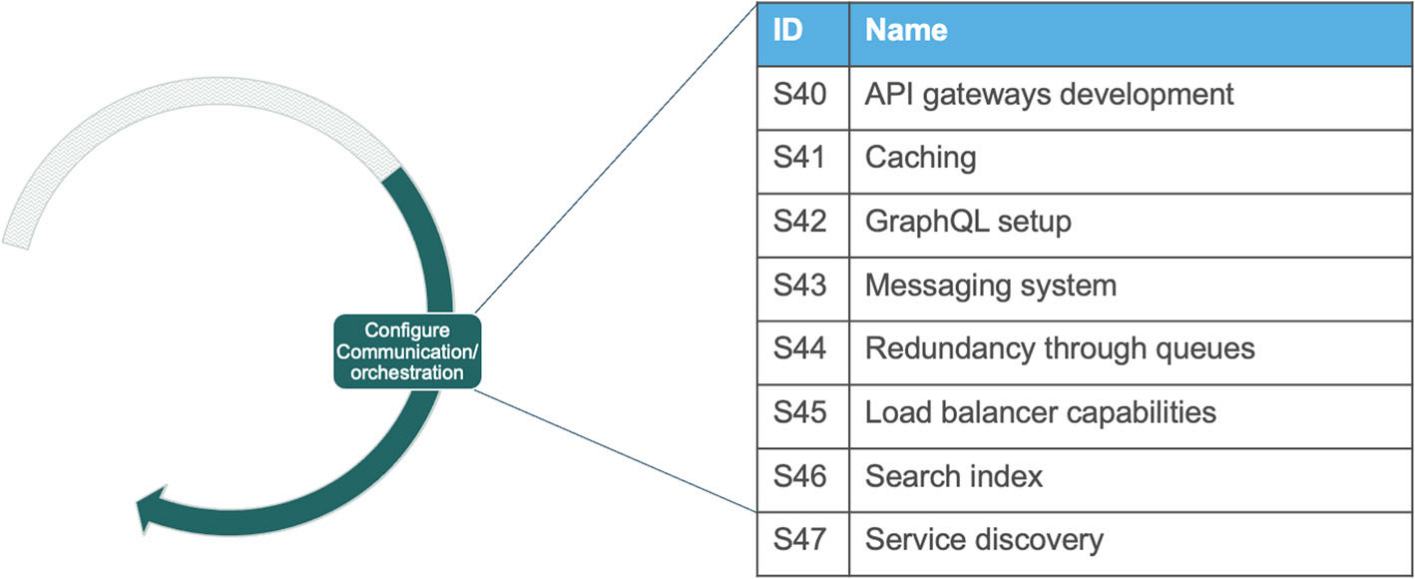
Solution outcomes for the activity “implement communication and orchestration capabilities”

A solution outcome identified (S40 in Fig. 16) is establishing communication via APIs that act as interface between services. As systems grow, such an outcome becomes a requirement in order to handle all communications between services. The second potential solution in this activity (S41 in Fig. 16) is caching in session-oriented connections, to keep temporarily data that might need to be used. Caching is a mechanism often used to implement the earlier solutions of having temporary data storage in services (i.e. S32) and a frontend database (i.e. S33). A suitable format that is used across engineers for exchanging data between communicating services is setting up GraphQL (S42). Another potential solution (S43 in Fig. 16) with high popularity is that of a messaging system that enables the publishing of event messages that await to be consumed. This often requires to have queuing systems that register event messages (S44). Also, another element in this paradigm is developing a load balancer (S45) that will help the scale out of applications through clusters of nodes. Search indexes (S46 in Fig. 16) can be used to access data from all services that need to be communicated. A reoccurring theme of these solution outcomes is their aim to achieve eventual consistency reliably, in order to maintain a more stateless and asynchronous flow. Finally, as the amount of microservices grows, is typically required to develop a way of finding them in an inventory and orchestrating their execution. The solution outcome identified for configuring communication and orchestration is to establish capabilities to process different requests that services do. This solution outcome aims at data requests processing and forwarding. For example, through setting up a load balancer (S44) that manages traffic and help to manage costs of servers. Also, it can be done with service discovery capabilities (S47 in Fig. 16). That is because when microservices are deployed independently and potentially in different and varied machines, a mechanism is required to automatically finding the right address to communicate with.

*“So eventual consistency is the only data consistency option in a microservices-based architecture, and if you need strong-consistency guarantees, then you need to build work-arounds (compensating operations), like retry flows, which will add additional complexity.”* - StackOverflow Discussion^18^

##### Execution challenges

- New tools and technologies (about communication and orchestration) that are introduced with microservices are often perceived as challenging to adopt and learn by the engineers Waseem et al. (2021). In addition, it is a challenge to assess the suitability of different technologies for services communication, without prior experience and attempts to design an optimal architecture.

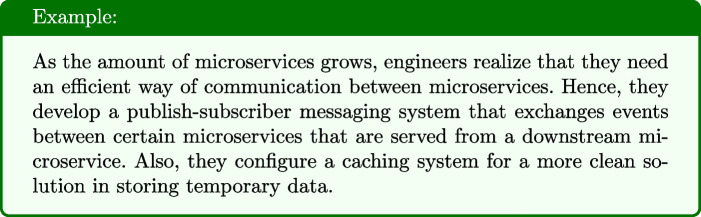

#### Setup Monitoring, Logging and Authentication Mechanisms

##### Context

Furthermore, a migration often includes the development of different *Monitoring, logging and authentication mechanisms*. This activity is about those cross-cutting concerns that appear in a distributed manner throughout the system, often affecting all microservices. The cross-cutting concerns take place in all services. These aspects have a different nature from other configurations (e.g., about setting up infrastructure) in the sense that they might affect the implementation as well (e.g. source code might need to be added for registering logs). For example, monitoring and logging have a different nature in MSAs, which needs to be designed accordingly. Specifically, interviewee I2 mentioned that putting proper exception handling to propagate errors correctly was not essential and entirely in place before. However, in microservices managing error messages and exceptions and propagating them to the entire system are vital for being able to locate issues and bugs inside a complex network of microservices.

##### Addressed problems

- This activity helps to isolate crucial aspects of governance that require specialized knowledge, introduced with microservices Wu et al. (2022).

**Fig. 17:**
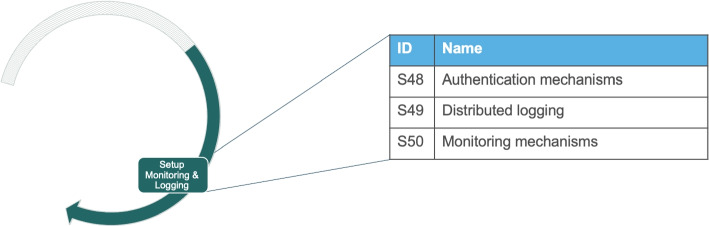
Solution outcomes for the activity “setup monitoring, logging and authentication mechanisms”

##### Solution outcomes

The solution outcome S48 in Fig. 17 of setting up an authentication mechanism is regarding developing a unified way in the application for users to gain access and navigate across services. We categorize authentication into 1) user authentication for managing users’ access, 2) microservices authentication for manage access from different features (e.g. internal/admin features have different access than external ones), and 3) authentication protocols for the governance of the system’s authentication and authorization. The solution outcome for distributed logging (S49 in Fig. 17) is regarding setting up distinctly bounded services that generate information on how they execute and communicate with each other in a transparent way, but without interfering with each other’s execution.

Finally, a solution for monitoring is crucial in order to have visibility on the faults and bugs that might happen in the application. The overall objective is to comprehensively keep track of shortcomings, especially in early stages of the migration.

*“Make sure you have a good setup for monitoring and alerts of any failure.”* - StackOverflow Discussion^19^

##### Execution challenges

- No specific and non-trivial challenges are identified for this activity, other than the fact that related tools and technologies are perceived novel and require a learning curve (Waseem et al. 2021).

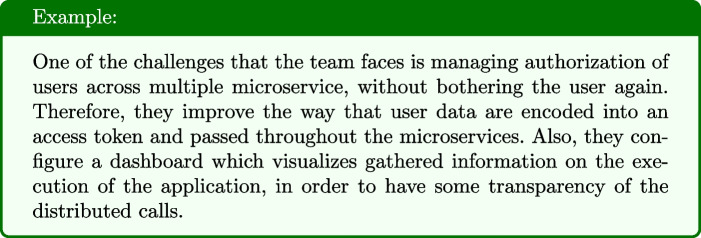

#### Handle Reused Artifacts

##### Context

Finally, more often than not, there are artifacts being reused from the old version of the system in a specific service. Hence, there is a need to design the ways for *handling any reused artifacts*.

##### Addressed problems

- This activity triggers the discussion on how legacy code is managed effectively, which is often challenging. Sometimes, a fully optimal solution that implements all principles from microservices might not be the most practical and realistic option, given its costs and expected benefits, and compromises need to be made. In addition, engineers need to find a way of maintaining a coherent architecture without disrupting functionality that is already working properly and for which there is no real benefit from migrating it to microservices.

##### Solution outcomes

**Fig. 18:**
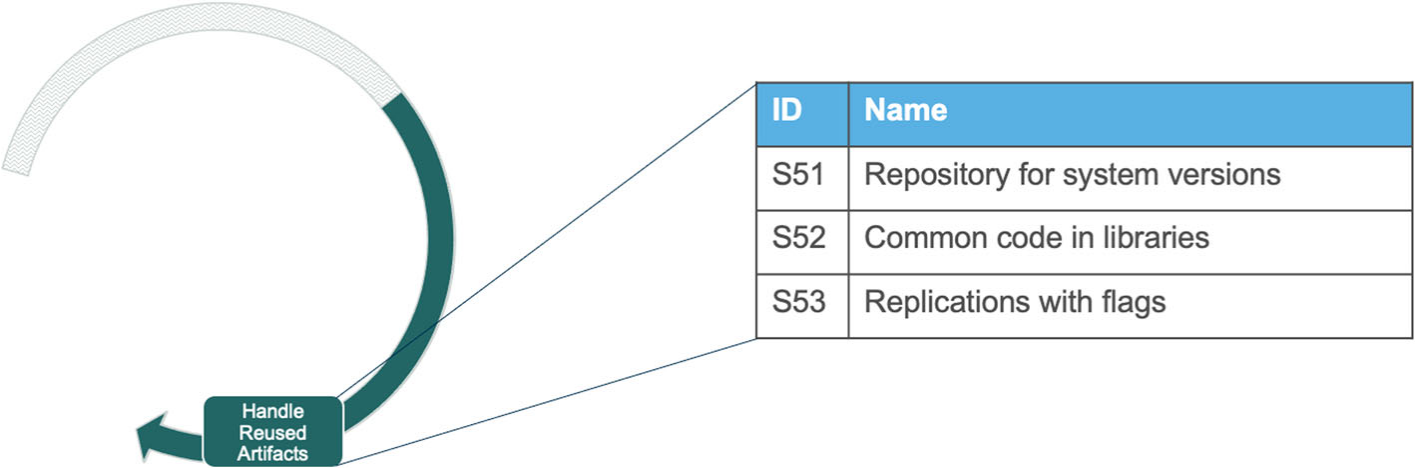
Solution outcomes for the activity “handle reused artifacts”

This happens for example, using libraries and APIs around smaller monolithic parts within the microservices. According to the analyzed discussions of engineers, this activity also includes the solution outcome of maintaining a repository that stores previous versions of the system’s parts (S51 in Fig. 18). For example, in order to navigate back to previous knowledge that might be useful in future versions.

The solution outcome (S52 in Fig. 18) of reusing technical artifacts via extracting common code to libraries is validated from the analyzed discussions. Also, the discussions revealed another solution for reusing technical artifacts. S53 is about creating replications of functionality with flags that indicate that they are in an intermediary stage of design.

##### Execution challenges

- In order to reuse artifacts practitioners will sometimes need to violate certain microservices principles, such as the principle of not using shared libraries. Choosing when to follow, and when to deviate from, best practices and microservices principles is non-trivial to decide.

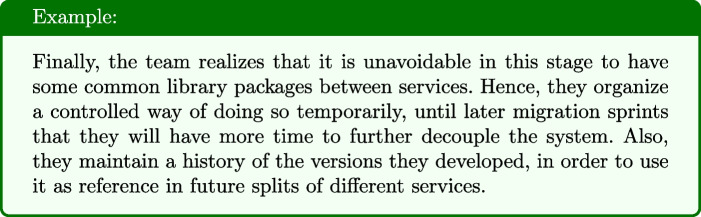

## Discussion

In our analysis we deconstruct migrations to their detailed elements. We provide a comprehensive picture of microservices migrations, with 2 connected modes of change that are pursued from different roles and responsibilities across a migrating organization. The migration journey entails a process of change, derived from empirical analysis (Ralph , 2019). In this study we identify the different elements that constitute a migration, showing how complex such an endeavour can be for the entire migrating organization. Another contribution, is that we start deciphering this complexity in a way that it is possible to indicate its constituent elements in the form of activities and solution outcomes. Our deconstruction of migrations is an important step to the direction of understanding and explaining migrations towards modern software architectures such as MSA.

### Theoretical Relevance and Future Work

Our study shows that microservice migration is complex including a multitude of activities and solutions. In the following, we discuss references that the reader can use to learn more about how the different activities can be executed. In addition, we discuss gaps and necessary future work, where our study revealed aspects of activities that are not yet detailed in related work. This discussion is summarized in Tables 4 and 5.

**Table 4:** Mapping activities of systemic migration with existing literature

*Activities*	*Existing work*	*Reference*	*Gap / Future work*
Clarify migration drivers	(Economic) benefits and impact (e.g. on TechDebt) of migrating to MSA	(Singleton , 2016; Fritzsch et al. , 2019; Lenarduzzi et al. , 2020; Taibi et al. , 2017)	Alignment between different backgrounds and concerns
Define criteria for decomposition	Decomposition approaches based on: size, teams, code, call traces, logic etc..	(Mazlami et al. , 2017; Fritzsch et al. , 2019)	Study metrics that can be used to inform a decomposition rationale in different stages of a migration
Build a shell API	Strangler fig pattern and service facade	(Newman , 2019; Knoche and Hasselbring , 2018)	Compare different ways that can implement the structuring of intermediary architectures until a mature MSA
Design a service cut	Services decomposition approaches (e.g. Service cutter)	(Gysel et al. , 2016; Di Francesco et al. , 2018)	Integrating engineers in service cut decisions
Setup continuous extraction	Process for extracting microservices and elements to consider that come with microservices	(Waseem et al. , 2021; Taibi and Lenarduzzi , 2018; Waseem et al. , 2021)	Transitioning cases towards stateless and understanding business roles in splitting services
Setup independent integration, testing and deployment	Mentioned need for additional development and configuration overhead for operations	(Soldani et al. , 2018; Waseem et al. , 2020)	Investigate how software engineers develop their skillset in the new DevOps paradigms

**Table 5:** Mapping activities of technical migration with existing literature

*Activities*	*Existing work*	*Reference*	*Gap / Future work*
Understand and analyze system	Investigate business logic, domain and potential existing solutions	(Newman , 2015; Newman , 2019; Hassan et al. , 2020)	Relate requirements engineering specifically about microservices migrations
Prepare backend	Formal model/guidance on how to track and split technical artifacts of the system	(Waseem et al. , 2021; Zhou et al. , 2021)	Investigate the process of designing architectural styles
Split Data	Distributed data management solutions and practices	(Waseem et al. , 2021; Ntentos et al. , 2019; Loukiala et al. , 2021)	Detail how practitioners migrate their data management solutions
Decompose frontend	General guidance on how frontend development takes place in microservices	(Newman , 2015)	Empirical investigation on the changing process of client-facing components of systems
Setup DevOps	Classification of DevOps problems, solutions and tools for MSA	(Waseem et al. , 2020)	Understand how developers change their engineering paradigms and mindset in microservices
Configure communication and orchestration	Lists of technologies that can be used for implementation of services communication and orchestration	(Jamshidi et al. , 2018; Zimmermann , 2017b; Waseem et al. , 2020)	Accumulate technology stack to achieve a new services communication paradigm
Setup monitoring and logging	Insights on the perspective of practitioners on artifacts complementing MSA	(Waseem et al. , 2021)	Investigate how to help practitioners adopt existing performance analysis practices from cloud computing
Handle reused artifacts	Taxonomy of anti-patterns and violations regarding coupling in microservices	(Taibi et al. , 2020; Ntentos et al. , 2021)	Determine what violations to Best Practices in MSAs can have positive impact

We identified those activities that have been discussed in the interviews and SO. However, it is possible that there are additional activities that we did not capture yet. For example, activities to validate the correctness and performance of the migrated system or activities to rollback to the old architecture, if the migration does not work well. Future work can extend the journey presented in this paper by uncovering such additional activities.

On the one hand, our results pave the way for researchers’ future work to use the derived modes of change for systematically reviewing existing literature that addresses the individual topics of activities. On the other hand, our contribution paves the way for future research in each activity to be studied in detail empirically for reaching more complete lists of solution outcomes in each step of a migration. Specifically, one future direction is to investigate in more detail what roles typically appear in microservices migrations and what are their responsibilities.

#### Systemic Migration

It is crucial to systemically re-architect the system in an iterative manner. Even though this is in-line with guidelines on decomposing microservices (Fritzsch et al. , 2018; Newman , 2019), there is room for more empirical investigations focusing on the gradual evolution of systems until they reach a MSA.

Current research describes benefits of migrating towards microservices, being technical or economic (Taibi et al. , 2017; Singleton , 2016). Our findings on the activity “clarifying migration drivers” enriches this current research with first insights on the need to align different backgrounds and concerns of stakeholders. This needs to be further studied in future work.

Furthermore, there are existing approaches, patterns, and processes to decompose services detailing on the activities “define criteria for decomposition”, “build a shell API”, “design a service cut”, and “setup continuous extraction” (Mazlami et al. , 2017; Fritzsch et al. , 2018; Knoche and Hasselbring , 2018; Taibi and Lenarduzzi , 2018; Newman , 2019; Gysel et al. , 2016; Waseem et al. , 2021; Michael Ayas et al. , 2021a). However, empirical evidence is required to further evaluate existing metrics to inform a decomposition rationale. Furthermore, there is still a need to compare different patterns to implement the structuring of intermediary architectures. Existing approaches for service decomposition focus on automating the decision instead of supporting engineers in their decision making. Hence, there is a gap in studying the human factors in designing service cuts. To the best of our knowledge there is also a lack of research on how to transform stateful systems parts to stateless services.

There are so far only little guidelines on designing additional technical artifacts and setting up Continuous Integration and Continuous Deployment capabilities (Waseem et al. , 2020; Balalaie et al. , 2016; Soldani et al. , 2018). Researchers can use the findings to investigate how software engineers develop their skillset in the new paradigm of development and operations. For example, there is room to empirically study the learning process of engineers that need to newly setup CI/CD pipelines.

#### Technical Migration

The activity and solution outcomes regarding “analyzing the system” and available practices can use existing research and the plethora of current tools and frameworks that describe practices for transitioning to microservices (Hassan et al. , 2020; Waseem et al. , 2020). However, there is still a gap in investigating what are the requirements for migrating towards a MSA.

There are some formal models on how to track and split technical artifacts of the system (Waseem et al. , 2021; Zhou et al. , 2021). However, even though there is knowledge about the two architectural styles, data request handler and data request listener, we do not know much about the process of implementing them.

Furthermore, there are existing solutions and practices for distributed data management (Loukiala et al. , 2021) that link to the activity of “split data”. However, there is still a need to further investigate what engineers specifically do when splitting their data models when migrating their data management solutions. Interestingly, one of the solutions identified in this study about combining shared and individual databases is not completely in line with existing literature on MSAs best practices (Newman , 2015). It is understandable why this takes place in intermediary designs, and this indicates the need to investigate further the intermediary designs of microservices migrations. In addition, researchers can start giving more attention in empirically investigating the changing process of client-facing components of systems, relating to the activity of “decomposing the frontend”.

There are classifications of DevOps problems, solutions and tools (Waseem et al. , 2020), lists of technologies that can be used for implementation of services communication and orchestration (Jamshidi et al. , 2018; Zimmermann , 2017b; Soldani et al. , 2018), and lists of artifacts that practitioners use when setting up monitoring and logging (Waseem et al. , 2021). However, many gaps are identified, especially on empirically understanding how engineers transform their software development paradigms and mindset in microservices. In addition, future research can accumulate all potential technologies that can be used to achieve a new services communication paradigm. Finally there is a need to investigate how microservice practitioners can adopt performance analysis practices that exist for cloud today.

Finally, we found that the way software engineers “handle reused artifacts” can be described as violations of best practices regarding coupling in microservices (Taibi et al. , 2020; Ntentos et al. , 2021) (e.g. through shared libraries). However, we believe that more research is needed to determine whether violations to existing best practices in MSAs can potentially have positive impact to the migration.

An interesting insight from our results is that setting up supporting artifacts often have an even more prominent role than other activities in a migration iteration, with four identified activities only for supporting artifacts (in comparison to four activities accounted for planning and execution of microservices decompositions).

### Modes of Change and the Reoccurring Phases of Migrations

#### Migration projects as continuous improvement initiatives

This study indicates the importance of the iterative and continuous nature of migrations. Specifically, we see that it is not a one-off project but a continuous endeavour that takes place in migration sprints. Many of the investigated software development teams that migrate do not consider the change of the system as a main value-adding project. Rather, they view the migration project as a necessary sideline activity and they focus on developing new features and value adding artifacts. Therefore, migrations are rather transformational and take place in parallel with other activities and thus, there is sometimes a pause and revisiting to the project. Furthermore, since it is such a complex and multidimensional endeavour, engineers need to keep in mind designing and developing for future updates and extensions.

Distinguishing the technical implementation work from the overall architectural and design activities can structure the work packages of engineers more effectively. To the best of our knowledge, current research does not account for such comprehensive views that indicate how different levels of abstractions in an organization connect to each other during a software architecture overhaul change. The identified modes of change and their details showcase how the long transformational journey that companies go through when migrating relate to the smaller technical projects of altering the software architecture. Future research can further investigate the connections between the technical and the systemic migration and explain what is the interplay between the two modes of change.

#### Understanding the progress of migration projects

One finding from our result is the importance of having visibility on the three phases of a migration sprint. This helps the engineers migrating to have a positional awareness of the progress. As many changes take place in organizations during migrations, there are different modes of change. We see how for different levels of the migration we have a different pace. It is challenging to know if sufficient progress is made and to demonstrate it and placing the migration in one of the phases makes it possible. Also this can be used to anticipate work ahead and avoid repetition of work or taking wrong directions that would generate the need for a lot of work later on. For example, completely neglecting logging and exception handling might not be a good idea if it is anticipated to be an activity later on. Furthermore, this can bring awareness that using microservices is not a silver bullet and has some flaws that is good to plan for.

### Implications for Engineering Teams

#### Diverse skills required

Based on our findings, engineers that started migrations had to educate themselves on the new technologies that microservices bring. This typically is taking place through the studying of Best Practices and available material regarding the technologies. Of course their goal was always to have a mature architecture that works in the ways that it is supposed to do. In addition, with microservices, concepts of individual service ownership are introduced to the engineering teams. Consequently, teams need to have more “T”-shaped abilities which means that more comprehensive and diverse skills are needed for each microservice. Such skills include developing different parts of the system, but also analysing business-wise the service, configuring tools and setting up the development or orchestration environments. Teams get in a position of designing the business and the software at the same time with development. Therefore, business-savvy programmers or programming-savvy Business Analysts and System Designers are needed in teams. This is often resolved by recruiting system architects or consulting services.

#### Shift of complexity from implementation to configuration

However, one of the most important realizations of teams is the shift from traditional development/programming to workflow design and configuration. This entails the shift of complexity from implementation-level to a level of communication amongst services. Sometimes, the lack of being up to speed with the right skills, but also the growth of the microservices, lead to a discrepancy between the design or intended development processes and activities from the actual ones that the engineers do in reality. For example, even though there is the perception that there is strong decoupling in microservices, in reality there is sometimes a chain of microservices that leads to dependencies and coupling, but on a different level of abstraction. Hence, there is a difference between the intended and actual structures and processes. As in any architectural migration, there are different constraints that have opposing implications and thus, trade-offs that need to be balanced. Hence, there is a need for setting up the prioritization rules in deciding between trade-offs.

### Answering the Research Questions

***RQ1****: How are microservice migrations structured?* One of the core insights of our study is the observation that there seems to be two separate but connected modes of change going on: the systemic and the technical migration. Both are driven by different roles in the organization and have a different focus and speed of a migration sprint. The systemic migration is driven by management, architects and senior engineers whereas the technical migration is driven by engineers, system administrators and team leaders. Nonetheless both modes of change are connected and influence each other. Decisions made in the systemic mode, will affect how the technical migration is performed. We further structured the two modes using three conceptual phases. Interestingly these phases are very similar for the two modes: Planning, Executing, setting up supporting artifacts. Future work can test our iterative model of conducting migrations to specific industries. Also, future work can try to separate which concepts are generally regarding the change that comes with migrations and which are specific to microservices migrations.

***RQ2****: What activities do the systemic and technical modes of change entail?* Activities in the systemic migration are concerned with clarification of drivers for the migration, but also definition of decision criteria which affect the technical migration, such as criteria for decomposition or criteria for the granularity of extraction. Besides that, the systemic migration entails activities for executing the migration and for building up an infrastructure to facilitate the technical migration. This includes infrastructures for integration, testing and deployment, logging and monitoring.

Activities of the technical migration utilize the infrastructure and decisions provided by the systemic migration. This includes activities to understand the business logic, handle migration in backend, data and frontend, setting up DevOps, testing, communication orchestration, and monitoring, as well as handling reused artifacts. Future work can investigate the specific activities with the aim to infer statistical correlations about their existence and their detailed relationships/sequence with each other.

***RQ3****: What common solutions are available to realize these activities?* Our study allowed us to put many known solutions into the context of the overall migration journey towards MSAs that are part of the migration activities. This way our results present an overview of how solutions are connected and contribute to the overall migration journey.

Future work can investigate any potential connections between different solution outcomes and different types of services or software. Specifically, we believe that practitioners can benefit from further procedures and guidelines that will map different types of services with common solutions to implement the identified activities for microservices migrations.

## Threats to Validity

We designed our research as a grounded theory study to empirically understand the phenomena taking place in migrations towards microservices. Our theory stems from empirical evidence and therefore, has a weight in its validity. However, some threats that are inherent to our chosen study methodology remain, which readers should take into consideration.

### External Validity

Specifically, we cannot claim representativeness of our study demographics for the software industry in general, as the sampled population for the interviews was mainly through our personal network and using a voluntary, opt-in procedure. To address and mitigate this threat, we selected interview participants that cover different sizes of companies, in different industries, and across different geographical regions. Another mitigation to this threat is the triangulation with discussions from StackOverflow, which are potentially from a broader population when it comes to different industries and company sizes. However, due to the selected platform, the posts are likely to reflect the voices of engineers stronger than the perspective of the management. This limitation might lead to missing solution outcomes in our results.

### Internal Validity

Furthermore, in terms of internal validity, an identified threat is that we are somewhat pre-exposed to existing research through our previous interest in the field. In addition, we also have practical experience and exposure to the extensive practitioner-focused guidance on how to conduct microservice migrations. This may have biased our interview design and analysis of the interviews and posts. In consequence some parts of the migration journey may have been given less prominence or judged as unimportant during analysis. For example, those not discussed in earlier work. However, we mitigate this issue by using interviews and posts to confirm the presence of activities. Activities that were reoccurring in both, interviews and posts, were more likely to be included, even if they have not been discussed in earlier works.

Another threat concerns the extraction of details about solution outcomes from the posted discussions, such as the characterization of solution types as well as migration stage. It is possible that the identified categories of types and migration stages are too coarse-grained and lack important distinctions. Future studies will be necessary to confirm or complete these categories. To mitigate the threat, that single data points did not include enough information for the analysis, we payed attention to analyse questions that received at least one answer and an overall positive score. Both choices were made to increase the likelihood the question itself will already contain necessary context information, as the StackOverflow community systematically rewards this type of questions over those that lack context.

Finally, a limitation of our study design is that we cannot claim that the identified journeys are the only way to successfully migrate towards microservices, but they are an aggregation of activities from many real-world migrations.

## Conclusion

MSAs are a popular contemporary architectural style incarnating modern service-oriented organizations (Zimmermann , 2017b). MSAs promise benefits from changing several aspects such as easier scalability, higher maintainability, improved time to market (Fritzsch et al. , 2019; Taibi and Lenarduzzi , 2018), or a natural way of transitioning to the cloud (Lin et al. , 2016). Most applications are not designed as MSAs from ground up and thus, migrations towards a MSAs are becoming popular. However, such migrations are complex and multidimentional (Michael Ayas et al. , 2021a). In this study, we dwell into the complexity of such migrations. We 1) give an overview of how they take place on a systemic and a technical migration and 2) give fine grained details of what activities and solution outcomes appear in MSA migrations.

Our analysis indicates the existence and connection between 2 different modes of change. Our 2 different modes of change present the architectural migration towards microservices from different perspectives. First, the perspective of managers, software architects and senior engineers that see the software from a macro level. Second, the perspective of engineers that see the migration from the specific technical steps that they have in their daily work.

In addition, we further describe in detail this journey with 14 high-level activities, that take place from groups of people within the migrating organization. In addition, we organize activities based on characteristics and in order to predispose development of supporting or additional technical artifacts. Finally, the level of detail in which migration activities are described provides an in-depth view of what actually happens in such a structural change.

We specify each activity with 53 concrete and detailed solution outcomes. The defined constituent elements of the migration process, across different levels of abstraction is a novel overview of how migrations towards microservices take place in an organization. The accumulation in a single big picture and further detailing them empirically contributes to the existing body of knowledge in understanding what are the actions of engineers in MSA migrations. We also address a Software Engineering research gap in inductively explaining phenomena of how software architectures are migrated from one state to microservices.

## Footnotes

1 https://meta.stackexchange.com/questions/289233/how-many-sites-are-there-on-stack-exchange

2 https://www.qsrinternational.com/nvivo-qualitative-data-analysis-software/home/

3 https://stackoverflow.com/questions/44949367/microservices-dependencies-and-events

4 https://stackoverflow.com/questions/55986736/how-to-create-a-short-readable-uuid-in-a-microservices-distributed-environment?rq=1

5 https://stackoverflow.com/questions/69482127/how-to-implement-read-side-across-multiple-microservices-or-modules-in-modular

6 https://stackoverflow.com/questions/45400096/migrating-multi-module-project-to-microservices

7 https://stackoverflow.com/questions/44949367/microservices-dependencies-and-events

8 https://stackoverflow.com/questions/43069858/orchestrated-vs-choreographed-service-oriented-architecture-in-large-scale

9 https://stackoverflow.com/questions/30449278/automation-of-releases-of-microservices-based-application

10 https://stackoverflow.com/questions/56696750/how-do-i-manage-micro-services-with-devops

11 https://stackoverflow.com/questions/64789123/how-to-design-a-microservice-that-needs-to-communicate-with-others

12 https://stackoverflow.com/questions/69360441/designing-event-based-architecture-for-the-customer-service

13 https://stackoverflow.com/questions/40377377/micro-services-communication

14 https://stackoverflow.com/questions/27007353/how-does-data-denormalization-work-with-the-microservice-pattern

15 https://stackoverflow.com/questions/54862355/data-replication-in-microservices/54864010

16 https://stackoverflow.com/questions/56585409/how-to-break-a-django-website-into-microservices/56586168#56586168

17 https://stackoverflow.com/questions/30449278/automation-of-releases-of-microservices-based-application/30452220

18 https://stackoverflow.com/questions/65217718/best-practice-to-make-client-handle-eventual-consistency-of-microservices/66852136

19 https://stackoverflow.com/questions/39063178/architecture-patterns-for-low-traffic-and-high-availability-web-application/39068799
